# Identification of Fusarium head blight sources of resistance and associated QTLs in historical and modern Canadian spring wheat

**DOI:** 10.3389/fpls.2023.1190358

**Published:** 2023-08-23

**Authors:** Kassa Semagn, Maria Antonia Henriquez, Muhammad Iqbal, Anita L. Brûlé-Babel, Klaus Strenzke, Izabela Ciechanowska, Alireza Navabi, Amidou N’Diaye, Curtis Pozniak, Dean Spaner

**Affiliations:** ^1^ Department of Agricultural, Food, and Nutritional Science, 4-10 Agriculture-Forestry Centre, University of Alberta, Edmonton, AB, Canada; ^2^ Morden Research and Development Centre, Agriculture and Agri-Food Canada, Morden, MB, Canada; ^3^ Department of Plant Science, University of Manitoba, Winnipeg, MB, Canada; ^4^ Department of Plant Agriculture, Crop Science Building, University of Guelph, Guelph, ON, Canada; ^5^ Crop Development Centre and Department of Plant Sciences, University of Saskatchewan, Saskatoon, SK, Canada

**Keywords:** association mapping, diversity panel, *Fusarium graminearum*, international wheat genome sequencing consortium, physical map, Western Canada

## Abstract

Fusarium head blight (FHB) is one the most globally destructive fungal diseases in wheat and other small grains, causing a reduction in grain yield by 10–70%. The present study was conducted in a panel of historical and modern Canadian spring wheat (*Triticum aestivum* L.) varieties and lines to identify new sources of FHB resistance and map associated quantitative trait loci (QTLs). We evaluated 249 varieties and lines for reaction to disease incidence, severity, and visual rating index (VRI) in seven environments by artificially spraying a mixture of four *Fusarium graminearum* isolates. A subset of 198 them were genotyped with the Wheat 90K iSelect single nucleotide polymorphisms (SNPs) array. Genome-wide association mapping performed on the overall best linear unbiased estimators (BLUE) computed from all seven environments and the International Wheat Genome Sequencing Consortium (IWGSC) RefSeq v2.0 physical map of 26,449 polymorphic SNPs out of the 90K identified sixteen FHB resistance QTLs that individually accounted for 5.7–10.2% of the phenotypic variance. The positions of two of the FHB resistance QTLs overlapped with plant height and flowering time QTLs. Four of the QTLs (*QFhb.dms-3B.1*, *QFhb.dms-5A.5*, *QFhb.dms-5A.7*, and *QFhb.dms-6A.4*) were simultaneously associated with disease incidence, severity, and VRI, which accounted for 27.0–33.2% of the total phenotypic variance in the combined environments. Three of the QTLs (*QFhb.dms-2A.2*, *QFhb.dms-2D.2*, and *QFhb.dms-5B.8*) were associated with both incidence and VRI and accounted for 20.5–22.1% of the total phenotypic variance. In comparison with the VRI of the checks, we identified four highly resistant and thirty-three moderately resistant lines and varieties. The new FHB sources of resistance and the physical map of the associated QTLs would provide wheat breeders valuable information towards their efforts in developing improved varieties in western Canada.

## Introduction

Fusarium head blight (FHB) is one the most globally destructive fungal diseases in wheat and other small grains. It is characterized by premature blighting of the spikes (plant death) and a reduction in grain yield by 10–70% due to shriveled kernels (Shah et al., 2018; Yi et al., 2018). FHB-infected grains are of unacceptable quality for marketing due to contamination with mycotoxins, which makes them not suitable for feed and food (Del Ponte et al., 2007). FHB is caused by several *Fusarium* species that may be present simultaneously in the same field. The prevalence of FHB species varies depending on geographical regions (temperate, sub-tropical, and tropical), weather conditions, the genetics of the varieties, and cultural practices (Waalwijk et al., 2003; Xu et al., 2005; Xu et al., 2008; Xue et al., 2019). Researchers from the Canadian Grain Commission identified a total of 13 *Fusarium* species in 454 durum (*Triticum turgidum subsp. durum (Desf.) Husn.*) and bread wheat (*Triticum aestivum* L.) samples collected in 1986-1987 of which six of the species accounted for 95% of the pathogen’s populations. *Fusarium avenaceum*, *F. acuminatum*, and *F. sporotrichioides* were the most prevalent species in both Manitoba and Saskatchewan, while *F. graminearum* was most prevalent in eastern Canada and southern Manitoba but was rarely observed in both Saskatchewan and Alberta (Clear and Patrick, 1990). A follow-up study confirmed the high prevalence of *F. graminearum* in eastern Canada, accounting for ~90% of nine fungal species identified in 492 spring wheat sampled between 2001 and 2017 in Ontario (Xue et al., 2019). In recent years, however, *F. graminearum* has become the predominant causal agent of FHB not only in eastern Canada but also in the three major wheat-producing Prairie provinces of Manitoba, Saskatchewan, and Alberta (Valverde-Bogantes et al., 2020; Bamforth et al., 2022). For example, Bamforth et al. (2022) studied a total of 7,783 durum and bread wheat samples originated from several farms between 2014 and 2020 in eastern and western Canada. Although their results showed a fluctuation in species abundance over the years, *F. graminearum* was the most abundant species in both eastern and western Canada and accounted for 75–95% of the total pathogen populations, followed by *F. avenaceum* (10%), and *F. acuminatum* (5%).

The most common strategies used to reduce the introduction, spread, and severity of FHB include varietal selection, planting clean seeds, seed treatment, increasing seeding rate, staggered planting dates among fields to avoid all fields from flowering at the same time, limiting irrigation before and during the flowering period, crop rotation, fungicide application, and use of biological controls (Bai and Shaner, 2004; Bergstrom et al., 2011). The development and planting of varieties with stable and durable resistance to FHB and mycotoxin accumulation have been frequently cited as the most economical and environmentally friendly approach for long-term control. However, the development of FHB-resistant varieties is more complicated for multiple reasons, including the presence of different mechanisms of resistance, the difficulty in identifying reliable sources of resistance germplasm with combinations of different mechanisms, the polygenic nature of FHB resistance (Buerstmayr et al., 2020), and the negative correlations between FHB resistance traits with plant height and flowering time (Miedaner and Voss, 2008; Skinnes et al., 2010; Lu et al., 2011; Lu et al., 2013; Buerstmayr and Buerstmayr, 2016; He et al., 2016; Ruan et al., 2020). Of the five types of FHB resistance mechanisms described in the literature (Schroeder and Christensen, 1963; Mesterházy, 1995), Type I (resistance to initial infection) and Type II (resistance to the spread of infection in the spike) are the two most widely studied forms of resistance (Hales et al., 2020). For that reason, the major focuses in the global FHB research involve the identification of new sources of Type I and Type II resistance, mapping of quantitative trait loci (QTLs), and developing FHB resistance germplasm by pyramiding multiple resistance alleles using conventional and/or modern breeding methods (Steiner et al., 2017; Buerstmayr et al., 2020; Mesterhazy, 2020; Zhang et al., 2021).

Most sources of FHB resistance are controlled by numerous QTLs with minor effects and a few QTLs with major effects (Venske et al., 2019; Singh et al., 2021). Two recent synthesis studies compiled a list of 556 QTLs (Venske et al., 2019) and 883 QTLs (Singh et al., 2021) associated with FHB resistance in several populations. Those QTLs were distributed in all 21 wheat chromosomes with each chromosome consisting of a cluster of numerous resistance QTLs. A meta-analysis conducted on 323 FHB resistance QTLs identified 56 meta-QTLs (Venske et al., 2019), which is a six-fold reduction in the initial number of QTLs. Such results suggest the possibility of reporting a cluster of the same QTLs as potentially novel. Although consensus genetic maps have been used to compare QTLs identified in different populations and independent studies, they are prone to substantial errors for multiple reasons, including a smaller number of common markers among populations for consensus map constructions, lack of physical positions for most types of markers, and genotyping errors (Zhang et al., 2020a). Most meta-QTLs were physically located within a short interval, which requires further refinement using the International Wheat Genome Sequencing Consortium (IWGSC) RefSeq (Zhu et al., 2021a) physical map. In previous studies, we have used the IWGSC physical positions of the Wheat 90K iSelect array (Wang et al., 2014) to characterize QTLs associated with agronomic traits and grain characteristics (Semagn et al., 2022b) and reaction to leaf rust, stripe rust, common bunt, and leaf spot (Iqbal et al., 2023) in a historical and modern Canadian spring wheat association mapping panel.

In western Canada, wheat diseases are divided into three priority groups with at least intermediate levels of resistance to priority-one diseases, which include FHB, stripe rust, leaf rust, stem rust, and common bunt (Brar et al., 2019b). FHB resistance QTLs were reported in biparental wheat populations derived from AAC Innova/AAC Tenacious (Dhariwal et al., 2020), FL62R1/Stettler and FL62R1/Muchmore (Zhang et al., 2018; Zhang et al., 2020b), DH181/AC Foremost (Yang et al., 2005), Vienna/25R47 (Tamburic-Ilincic and Barcellos Rosa, 2017), and Maxine/FTHP Redeemer (Tamburic-Ilincic and Rosa, 2019). Some of the issues of QTLs discovered in biparental populations include (i) poor resolution due to a limited number of recombination events, (ii) only alleles originating from two parents used in developing a given population are captured, and (iii) high multicollinearity among pairs of polymorphic markers in each population, which results to the exclusion of markers that differ by< 1 cM in the final data analyses (Semagn et al., 2006; Wang et al., 2006). Genome-wide association study surveys historical recombination frequencies in diverse genetic backgrounds and unrelated pedigrees developed for a wide range of purposes. It tend to be superior to linkage-based QTL mapping in biparental populations by testing genetic variants across the whole genome and finding genotypes statistically associated with phenotypes in unrelated individuals (Uffelmann et al., 2021). This methodology was used to map QTLs associated with FHB resistance in a Canadian durum wheat association mapping panel (Ruan et al., 2020) and durum wheat breeding lines assembled from 72 diverse crosses (Sari et al., 2020). We are not aware of previous GWAS to map QTLs associated with FHB resistance in historical and modern Canadian spring wheat varieties. The objectives of this study were, therefore, to (i) map QTLs associated with FHB incidence, severity, and visual rating index using genome-wide association analysis and the IWGSC RefSeq v2.0 physical map of the Wheat 90K iSelect array, (ii) understand if the positions of FHB resistance QTLs overlaps with QTLs for flowering time and plant height, and (iii) identify new sources of FHB resistance in historical and modern Canadian spring wheat varieties and lines.

## Materials and methods

We used a total of 249 spring wheat varieties and lines adapted to the western Canada growing conditions (**Supplementary Table S1**), which included 200 historical and modern varieties registered for commercialization in Canada from 1905 to 2022, 47 unregistered lines developed by Canadian breeders, and 2 exotic lines (Sumai 3 and Saar). Sumai 3 (Yong-Fang et al., 1997) is one of the most widely studied Chinese cultivars, while Saar is a line developed by the International Maize and Wheat Improvement Center (Lillemo et al., 2008) and characterized by a moderate level of resistance to FHB (Wiśniewska et al., 2016). AC Vista and CDC Teal were used as FHB susceptible checks, both AC Cora and AC Barrie as intermediately resistant checks, 5602HR as a moderately resistant check, and both Sumai 3 and FHB37 as highly resistant checks. FHB37 is an experimental line developed from the cross HY611/Ning8331 and has the same *Qfhb-5AS* allele as Ning 8331 derived from Sumai 3 (Pandurangan et al., 2020).

Reactions to *F. graminearum* were evaluated at seven environments in eastern and western Canada, which included the Elora Research Station, Pilkington, Ontario in 2017 (Elora-2017), the Ian N. Morrison Research Farm, Carman, Manitoba in 2020 (Carm-2020), and the Morden Research and Development Center, Morden, Manitoba from 2017-2019 and 2021-2022 (Mord-2017, Mord-2018, Mord-2019, Mord-2021, and Mord-2022). To minimize the confounding effects of other wheat diseases, reactions to FHB were evaluated within 13 acres of land dedicated to *F. graminearum* screening. In addition, we also planted two meters wide border rows around the FHB nurseries to minimize infection by stray pathogens with tools, vehicles, and field equipment exclusively assigned to the FHB nursery. The detailed FHB evaluation method has been described in a previous study (Semagn et al., 2022a). Briefly, a suspension of four *F. graminearum* isolates at a concentration of 50,000 macroconidia mL^-1^ was prepared by mixing an equal amount of two 3-ADON (HSW-15-39 and HSW-15-87) and two 15-ADON (HSW-15-27 and HSW-15-57) chemotypes with sterile water and Tween 20 (Dhariwal et al., 2020). Wheat spikes were sprayed using a backpack sprayer when about 50% of the plants within a plot reached flowering. Inoculation was repeated 2-3 days later to infect tillers with delayed flowering time. Inoculated plants were irrigated three times weekly using an overhead mist irrigation system or Cadman Irrigations travelers with Briggs booms.

Disease incidence (the proportion of diseased plants) and severity (the area of plant tissue that was visibly diseased) were recorded on a scale of 0 (highly resistant) to 10 (highly susceptible) in three environments (Mord-2017, Mord-2018, and Mord-2019) and from 0 to 100% at the remaining four environments (Elora-2017, Carm-2020, Mord-2021, and Mord-2022) after 18-21 days from the first inoculation. To get the same disease rating in all environments, however, we converted the percent scores into a 0 to 10 scale. Visual rating index (VRI) was calculated by multiplying disease incidence and severity recorded into a 0 to 10 scale as described in a previous study (Gilbert and Morgan, 2000). Flowering time was recorded as the number of days from planting to the emergence of a few anthers in the middle of spikes at three environments (Mord-2017, Mord-2018, and Mord-2019). Plant height was measured from the base of the stem to the tip of the terminal spikelet excluding the awns at five environments (Mord-2017, Mord-2018, Mord-2019, Mord-2021, and Mord-2022).

The 249 lines and varieties evaluated for reaction to FHB were genotyped with Wheat 90K iSelect in two sets. The first set of 203 lines and varieties was genotyped at the University of Saskatchewan, Saskatoon, Canada in 2018. The remaining 46 lines and varieties were genotyped along with other samples at the Agriculture and Agri-Food Canada (AAFC) lab in Morden. All samples were also genotyped with 14 Kompetitive Allele-Specific PCR (KASP) markers associated with *Fhb1* (wMAS000008, wMAS000009, BS00003814, BS00009393, BS00009992, and BS00012531), *Rht-B1* (wMAS000001), *Rht-D1* (wMAS000002), *Glu-A1* (wMAS000012 and wMAS000013), *Glu-D1* (wMAS000014), *Lr34* (wMAS000003 and wMAS000004), and *Sr2* (wMAS000005). KASP genotyping was done using the Biosearch Technologies (https://www.biosearchtech.com/; accessed on 15 Feb 2023) service lab, Beverly, MA, USA. However, merging of the 90K SNPs genotype datasets generated by the University of Saskatchewan and AAFC labs was problematic due to inconsistencies in allele calls of the positive controls (Carberry, Glenn, and Park), and differences in the total number of SNPs successfully called by in both datasets (24,926 of the 90K SNPs by AAFC vs. 55,404 SNPs by the University of Saskatchewan). Challenges in merging SNP data generated by different labs and groups are a common constraint for different reasons, including strand orientation (Zuvich et al., 2011; Verma et al., 2014). Therefore, we only used the genotype data of 198 of the 203 lines and varieties after excluding five samples that had high missing genotype data and/or residual heterozygosity.

Of the 90K SNP array and KASP markers used to genotype the 198 lines and varieties, we used 26,449 polymorphic SNPs (**Supplementary Table S2**) for statistical analysis after removing markers with a minor allele frequency of<5%, heterozygosity of >20%, missing data of >30%, and those with unknown chromosomes based on the IWGSC RefSeq v2.0 (Zhu et al., 2021a). Sequences of the polymorphic SNPs were retrieved from the GrainGenes database (https://wheat.pw.usda.gov/GG3/) and blasted against the IWGSC RefSeq v2.0 in the Wheat@URGI portal (http://wheat-urgi.versailles.inra.fr/Seq-Repository/BLAST). The default blast parameters were used, followed by filtering based on the best scores (Okada et al., 2019). The final genotype data was imputed using LinkImpute implemented in TASSEL v5.2.86 (Bradbury et al., 2007).

Multi Environment Trial Analysis with R (META-R) v.6.04 (Alvarado et al., 2020) was used to compute the best linear unbiased predictors (BLUP), best linear unbiased estimators (BLUE), genetic and phenotypic correlation coefficients, and variance component analyses. Broad-sense heritability (H^2^) in the combined environments and repeatability between replications within each environment (H) were computed from the variance components as follows:

${\text{H}}^{2}=\frac{\sigma^{2}{\;}_{g}}{\sigma^{2}{\;}_{g}+\frac{\sigma^{2}{\;}_{\mathrm{ge}}}{\text{nEnv}}+\frac{\sigma^{2}{\;}_{\varepsilon }}{\text{nEnv x nRep}}}$ and $\text{H}=\frac{\sigma^{2}{\;}_{g}}{\sigma^{2}{\;}_{g}+\frac{\sigma^{2}{\;}_{\varepsilon }}{\text{nRep}}}$

where σ^2^_g_, σ^2^_ge_, σ^2^_ε_, nEnv, and nRep refer to genotypic variance, G×E interaction variance, residual error variance, number of environments, and number of replications, respectively (Alemu et al., 2021). We used JMP v16 statistical software (Jones and Sall, 2011) for coefficients of determination (R^2^) analysis and to generate different types of graphs from the phenotype data. Using the Prairie Recommending Committee for wheat, rye, and triticale operating protocol (PRCWRT, 2020), we assigned lines and varieties into five groups by comparing their overall VRI computed from all seven environments with checks as follows: highly resistant (VRI< 7.0), moderately resistant (7–20.0), intermediate (20.1–30.0), moderately susceptible (30.1-40.0), and highly susceptible (VRI > 40.0). These ranges were modified from previous threshold values described in Chinese wheat germplasm: highly resistant (VRI< 10%), moderately resistant (10–25%), moderately susceptible (25–45%), and highly susceptible (VRI > 45%) (Yan et al., 2020; Yan et al., 2022).

Identity by state (IBS)-based genetic distance matrices and principal component analysis (PCA) were computed using TASSEL v5.2.86 (Bradbury et al., 2007). Phylogenetic trees were constructed from the IBS-based distance matrices using the neighbor-joining method implemented in Molecular Evolutionary Genetics Analysis (MEGA) v11 (Tamura et al., 2021). Scatter plots were generated as an indicator of the population structure in the germplasm by plotting the first three principal components from PCA in CurlyWhirly v1.21.08.16 (The James Hutton Institute, Information & Computational Sciences). Marker trait associations (MTA) were identified using the weighted mixed linear model method implemented in TASSEL. The input data consisted of the imputed SNP genotype data, a kinship matrix to account for relatedness, PC1 to PC3 from a principal component analysis to account for population structure and BLUEs of each trait computed within each environment and combined across all environments. The number of polymorphic SNPs used in the final analysis varied from 307 on chromosome 4D to 2,276 on 2B (**Supplementary Table S2**) with an overall average of 1,259 SNPs per chromosome. The BLUEs used for marker-trait association analyses included disease incidence, severity, and VRI as the primary traits. Flowering time and plant height were used to explore if any of the SNPs significantly associated with disease incidence, severity, and VRI were also simultaneously associated with these two agronomic traits. Markers were declared as significant at a false discovery rate of *p*< 3.1 × 10^−4^ or -log_10_ (p) value of ≥ 3.5. Genome-wide Manhattan plots were obtained using SNPevg (Wang et al., 2012), while quantile-quantile (QQ) plots were generated in TASSEL. Two or more adjacent SNPs significantly associated with the same trait that differs by less than 15 Mb were assigned to the same QTL designation using a trait acronym (Fhb, Flt, or Pht), lab designation (dms = Dean Michael Spaner), and chromosome number. The positions of QTLs that consisted of a cluster of two or more SNPs significantly associated with each trait were given as an interval using the minimum and maximum positions. QTLs associated with two or more traits but differ by<15 Mb were considered coincident. QTL map of each chromosome was constructed using MapChart v2.32 (Voorrips, 2002).

## Results

### Phenotypic variation

**Figure 1:**
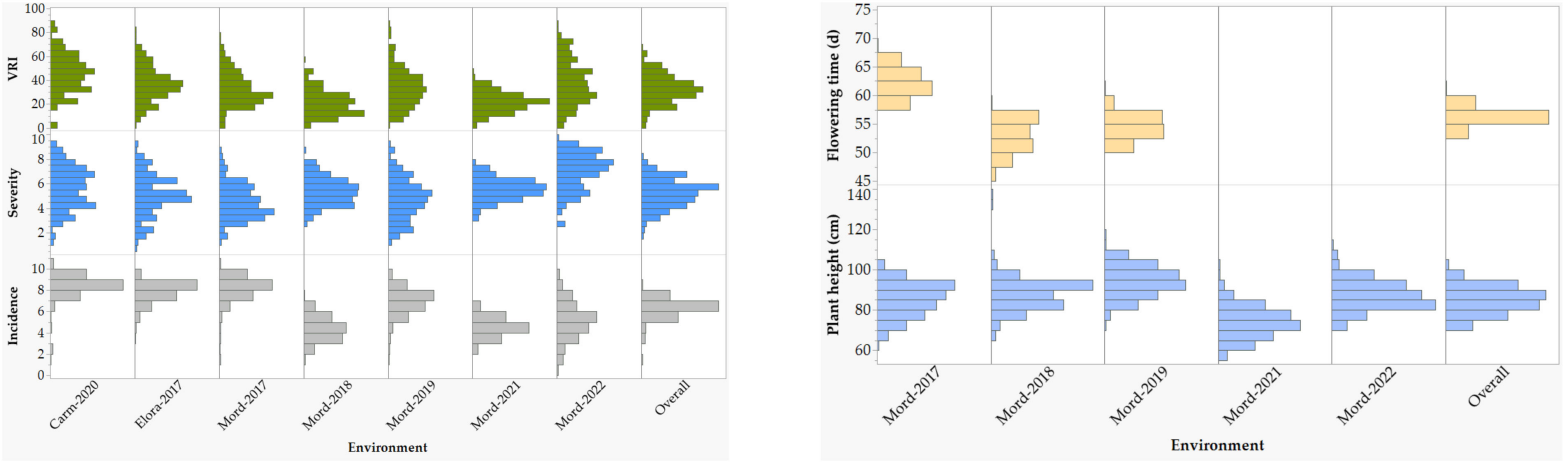
Frequency distributions of reactions to Fusarium head blight (FHB) incidence, severity, and visual rating index of 249 spring wheat varieties and lines evaluated at seven environments: the Ian N. Morrison Research Farm in Carman (Carm-2020), the Elora Research Station (Elora-2017), and the Morden Research and Development Center (Mord-2017, Mord-2018, Mord-2019, Mord-2021, and Mord-2022). Plant height and flowering time were evaluated at five and three environments, respectively. For each trait, the overall refers to the means of all combined environments.

The coefficients of determination (R^2^) between BLUPs and BLUEs computed for each trait within each environment and combined environments were very high for disease incidence, severity, and VRI, which varied from 0.96 to 1.00 (**Supplementary Figure S1**). As a result, all the subsequent results are based on the BLUEs. The overall mean BLUE values of disease incidence, severity, VRI, flowering time, and plant height computed in each environment varied from 0.5 to 10, 0.5 to 9.9, 0.5 to 89.9%, 47 to 69 days, and from 54 to 120 cm, respectively. Disease incidence, severity, and VRI of the 249 varieties and lines recorded in individual and combined environments showed continuous distribution in each environment but tend to be less severe at Morden both in 2018 and 2021 than in all other environments (**Figure 1**; **Supplementary Table S1**). The Shapiro-Wilk tests for normality performed on individual environments showed significantly skewed (p< 0.05) distributions for most trait-environments combinations, except the VRI both at Carm-2020 and Elora-2017, plant height at Mord-2018, Mord-2019, and Mord-2021. However, the distributions of mean BLUEs computed from all combined environments were normal or nearly so for all five traits. In the combined analyses of all environments, mean disease incidence, severity, VRI, flowering time and plant height ranged from 1.8 to 8.8, 1.9 to 8.2, 1.6 to 69.1%, 54 to 62 days and 67 to 104 cm, respectively (**Supplementary Table S1**).

**Figure 2:**
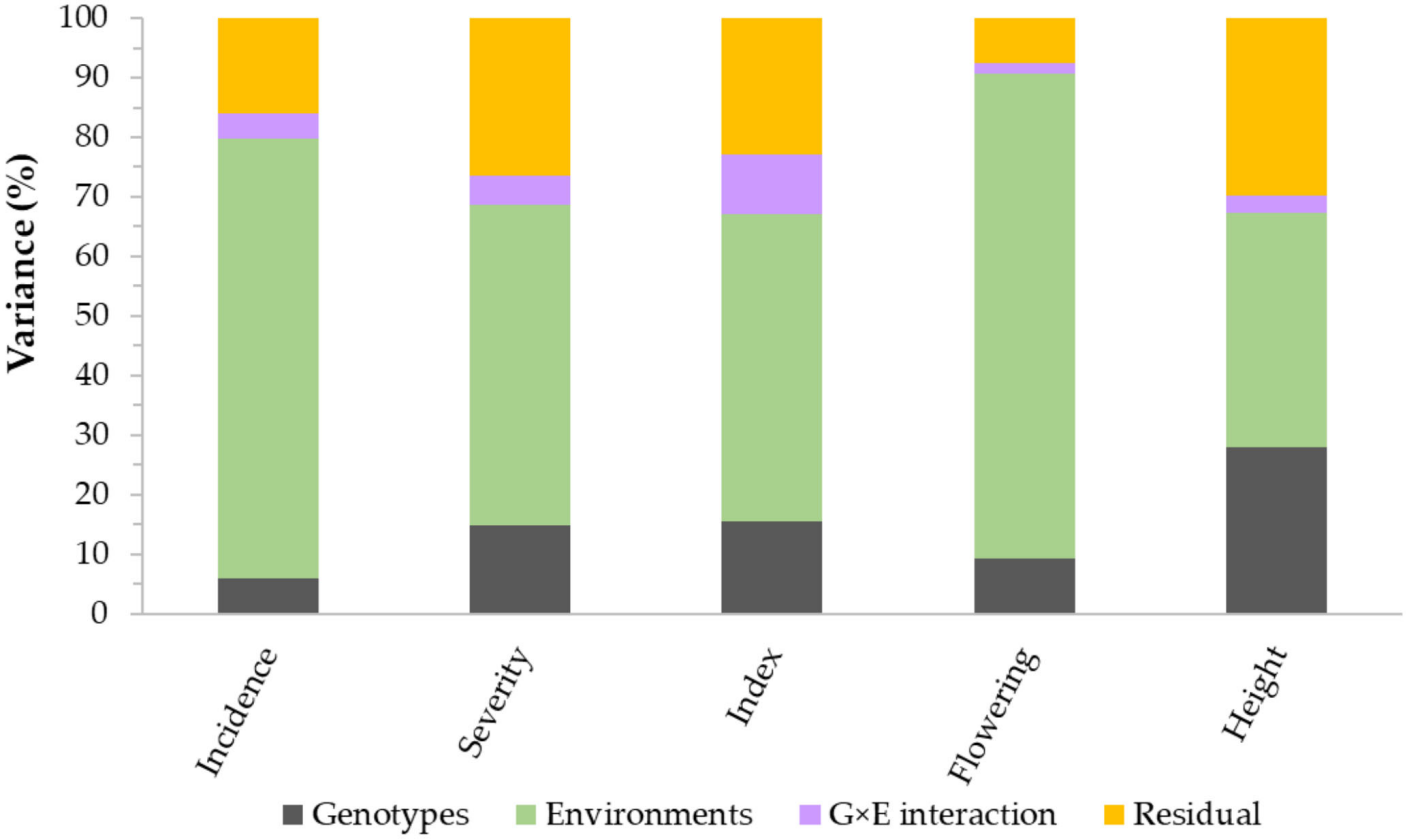
Partitioning of the total variance of genotypes (G), environments (E), G×E interaction, and residual error variances. The plot is based on 249 spring wheat varieties and lines evaluated for Fusarium head blight (FHB) incidence, severity, and visual rating index at seven environments, plant height at five environments, and flowering time at three environments.

Analysis of variance performed across all environments revealed highly significant (p< 0.01) differences among lines/varieties, environments, and G×E interaction (**Table 1**). However, differences among environments accounted for 39.3–81.4%, which is up to 12-fold greater than the genotypic variance. G×E interactions and residual error variances accounted for 1.7–10.0% and 7.6–29.7% of the total variances, respectively (**Figure 2**). Broad-sense heritability computed from all environments varied from 0.79 for disease incidence to 0.90 for plant height (**Table 1**). Repeatability computed between replications within each environment varied from 0.42 to 0.76 for disease incidence, 0.44 to 0.86 for disease severity, 0.50 to 0.89 for VRI, 0.41 to 0.90 for plant height, and 0.69 to 0.98 for flowering time (**Supplementary Table S3**). For each trait, we observed highly variable genetic correlation coefficients between pairs of environments, which ranged from 0.35 to 0.97 for the three FHB resistance traits, 0.24 to 0.84 for flowering time, and 0.77 to 0.98 for plant height. Phenotypic correlation coefficients between pairs of environments varied from 0.16 to 0.69 for the three FHB resistance traits, 0.48 to 0.77 for plant height, and 0.22 to 0.65 for flowering time (**Supplementary Table S3**). The coefficients of determination between pairs of traits within each environment varied from 0.05 to 0.48 between disease incidence and severity, 0.19 to 0.83 between incidence and VRI, and 0.52 to 0.93 between severity and VRI (**Supplementary Figure S2**). In the combined data of all seven environments, the overall correlations were 0.48 between disease incidence and severity, 0.68 between incidence and VRI, and 0.91 between severity and VRI. Both flowering time and plant height showed very small R^2^ with disease incidence, severity, and VRI (0.01< R^2^< 0.11).

**Table 1:** Variance components of Fusarium head blight incidence, severity, visual rating index, plant height, and flowering time recorded at 3–7 environments.

Statistics	Disease incidence	Disease severity	Visual rating index	Flowering time	Plant height
Genotype variance (σ^2^g)	0.54	0.93	82.40	3.08	38.21
Environment variance (σ^2^e)	6.61	3.36	274.66	26.86	53.47
Genotype × environment interaction (σ^2^ge)	0.38	0.30	53.24	0.55	3.91
Residual error variance (σ^2^ε)	1.43	1.66	121.37	2.49	40.42
Grand mean	5.72	4.25	27.77	58.30	85.04
Least significance difference (LSD)	1.03	1.12	11.08	2.34	6.26
Coefficient of variation (CV)	20.94	30.32	39.68	2.71	7.48
Mean number of replicates	2.29	2.29	2.29	2.50	2.40
No. of environments	7	7	7	3	5
P value for genotypes	< 0.01	< 0.01	< 0.01	< 0.01	< 0.01
P value for environments	< 0.01	< 0.01	< 0.01	< 0.01	< 0.01
P value for G×E interaction	< 0.01	< 0.01	< 0.01	< 0.01	< 0.01
Broad-sense heritability	0.79	0.86	0.84	0.80	0.90

### FHB resistance QTLs

The first three principal components (PCs) from a principal component analysis accounted for 24.0% of the total variation. A plot of the PC1 (11.7%), PC2 (7.5%), and PC3 (4.8%) revealed two major groups based primarily on kernel hardness/texture, gluten strength, and/or grain protein content (**Supplementary Figure S3** and **Supplementary Table S1**). Varieties and lines with medium to hard kennels and high grain protein content (> 11% measured as N x 5.7 on a 13.5% moisture basis) formed the first group, which included CWRS, CNHR, and CWHWS classes. Lines and varieties with soft and medium hard kernels (CPSR, CPSW, CWSP, and CWSWS) plus hard kennels with extra strong gluten strength (CWES) formed the second group. Details on the extent of molecular diversity, relatedness, linkage disequilibrium, and population structure of the diversity panel have been described in a previous study (Semagn et al., 2021). Genome-wide association analyses performed on the BLUEs computed within each environment and overall means of all combined environments identified a total of 559 significant marker-trait associations (MTAs). Of these 559 SNPs, 394 SNPs were associated with FHB resistance, six SNPs with FHB and plant height, one SNP with FHB and flowering time, 67 SNPs with flowering time, and 91 SNPs with plant height. One of the 559 SNPs located at 760.0 Mb on chromosome 2A (wsnp_Ex_c2137_4014383) was associated with both FHB severity and flowering time. Six SNPs located at 575.2 Mb on 2D (BobWhite_c17572_339), 379.7 Mb on 3A (wsnp_Ex_c22766_31972812), 597.6 Mb on 6A (both BobWhite_c14882_143 and Excalibur_c18632_1700), and 14.9 on 7B (BobWhite_c4253_568 and Excalibur_c34807_206) were associated with disease incidence and plant height.

**Figure 3:**
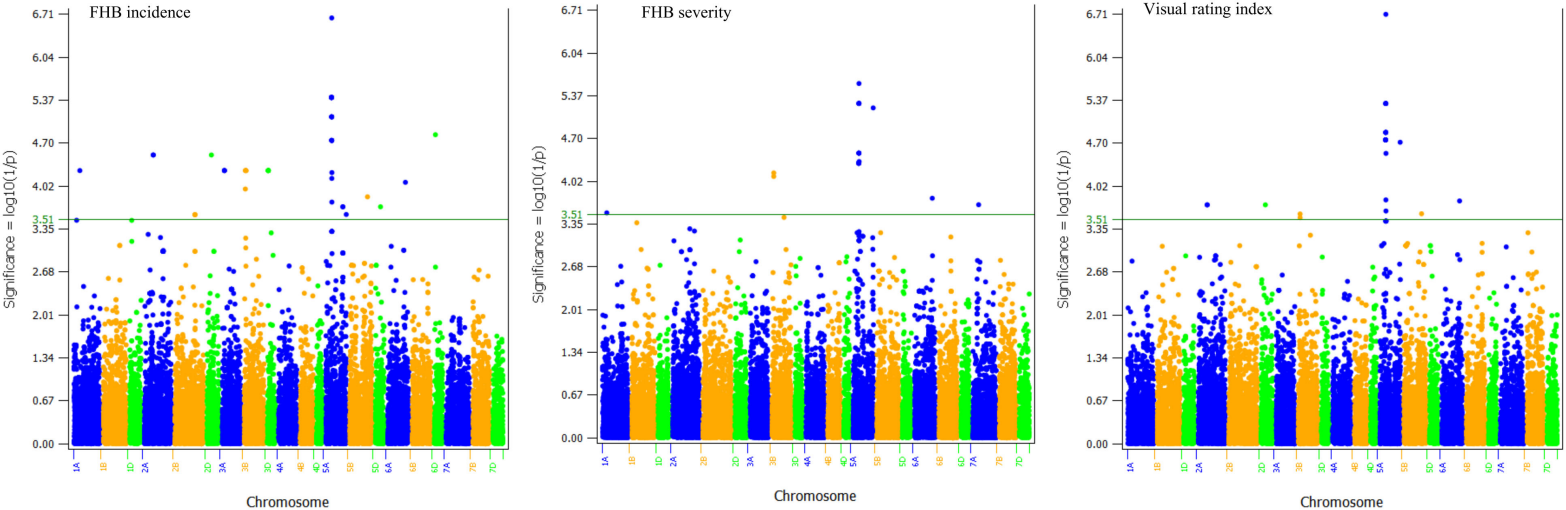
Genome-wide p values of 26,449 polymorphic SNPs based on a weighted mixed linear model and the best linear unbiased estimators (BLUEs) of Fusarium head blight (FHB) incidence, severity, and visual rating index computed from all seven environments. The horizontal line shows the threshold p-value of 3.1×10^-4^ or Log10 (1/p) value of 3.51. The A, B, and D genomes are in blue, orange, and green colors, respectively. Chromosomes and physical positions are shown on the x-axis.

**Figure 4:**
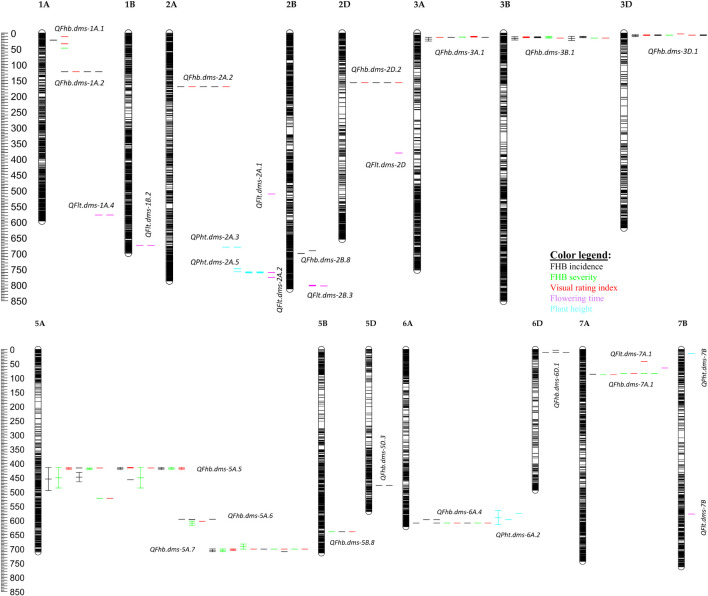
Visual rating index of seven checks, four highly resistant, and thirty-three moderately resistant varieties and lines based on overall mean best linear unbiased estimators (BLUE) computed from all seven environments. See **Supplementary Table S1** for details.

The 559 SNPs were clustered into 185 QTLs and were associated with plant height (37 QTLs), flowering time (36), disease incidence (38), severity (12), VRI (12), incidence and severity (1), incidence and VRI (7), severity and VRI (14), and disease incidence, severity and VRI (28). The 185 QTLs were distributed in all 21 wheat chromosomes, each consisting of 1a cluster of up to 27 significant SNPs, and individually accounted for 6.1–15.9% of either the individual or combined environments (**Supplementary Table S4**). However, only 75 out of the 559 SNPs (**Figure 3**) and twenty-eight out of the 185 QTLs (**Table 2**; **Figure 4**; **Supplementary Figure S4**) were identified using BLUEs computed from all combined environments, which included sixteen FHB resistance, four plant height, and eight flowering time QTLs. Below, we provided detailed results of only the 28 QTLs identified in the overall phenotype data of all environments and their expression in individual environments.

**Table 2:** Summary of QTLs associated with Fusarium head blight incidence (Inc), severity (Sev), visual rating index (VRI), plant height (Pht), and flowering time (Flt) associated with individual and combined environments.

QTL	No. of SNPs	Chr	Position (Mb)	P-value	PVE (%) in overall environments*					PVE (%) in individual and combined environments				
					Inc	Sev	VRI	Pht	Flt	Inc	Sev	VRI	Pht	Flt
*QFhb.dms-1A.1*	4	1A	12.4-48.8	1.8×10^-4^		6.5				7.6	6.5	6.9		
*QFhb.dms-1A.2*	1	1A	123.9	4.8×10^-5^	7.1					7.6		7.7		
*QFhb.dms-2A.2*	2	2A	170.8	8.0×10^-5^	7.6		6.9			8.0		7.0		
*QFhb.dms-2B.8*	4	2B	691.1-700.1	2.1×10^-4^	6.7					6.0				
*QFhb.dms-2D.2*	1	2D	159.1	8.0×10^-5^	7.6		6.9			8.0		7.0		
*QFhb.dms-3A.1*	9	3A	10.5-26.0	1.1×10^-4^	7.1					7.0	8.0	7.9		
*QFhb.dms-3B.1*	16	3B	10.2-23.5	9.9×10^-5^	7.0	7.7	6.5			7.0	10.2	7.9		
*QFhb.dms-3D.1*	9	3D	3.1-11.7	6.9×10^-5^	7.1					7.5	7.8	7.6		
*QFhb.dms-5A.5*	33	5A	414.2-522.7	6.9×10^-5^	7.7	8.7	9.2			7.5	8.4	9.1		
*QFhb.dms-5A.6*	11	5A	596.2-617.1	8.5×10^-5^	5.9					7.8	7.8	9.5		
*QFhb.dms-5A.7*	5	5A	683.3-710.1	1.1×10^-4^	5.7	9.8	8.9			6.6	8.6	9.2		
*QFhb.dms-5B.8*	1	5B	640.0	2.1×10^-4^	6.2		6.6			6.2	6.8	6.6		
*QFhb.dms-5D.3*	1	5D	477.8	1.3×10^-4^	5.9					6.5				
*QFhb.dms-6A.4*	5	6A	597.6-609.4	9.2×10^-5^	6.7	6.9	7.0			7.5	8.4	7.0		
*QFhb.dms-6D.1*	2	6D	3.5-12	7.6×10^-5^	7.2					8.2				
*QFhb.dms-7A.1*	5	7A	43.0-89.2	1.6×10^-4^		6.7				6.9	7.3	6.6		
*QPht.dms-2A.3*	11	2A	679.8-680.0	1.1×10^-4^				7.2					7.4	
*QPht.dms-2A.5*	3	2A	748.8-762.1	1.3×10^-4^				7.0					7.5	
*QPht.dms-6A.2*	14	6A	565.7-615.3	1.4×10^-4^				7.1					7.5	
*QPht.dms-7B*	2	7B	14.9	2.3×10^-4^				6.7					6.7	
*QFlt.dms-1A.4*	1	1A	579.0	4.3×10^-5^					8.3					8.0
*QFlt.dms-1B.2*	1	1B	675.7	9.2×10^-5^					7.6					7.3
*QFlt.dms-2A.1*	1	2A	511.5	1.3×10^-4^					6.9					6.9
*QFlt.dms-2A.2*	2	2A	760.1-776.7	2.9×10^-4^					6.1					6.1
*QFlt.dms-2B.3*	2	2B	800.7-803.2	8.0×10^-5^					8.2					7.9
*QFlt.dms-2D*	1	2D	382.6	7.4×10^-5^					7.4					7.4
*QFlt.dms-7A.1*	1	7A	65.8	2.4×10^-4^					6.4					6.4
*QFlt.dms-7B*	1	7B	578.2	2.2×10^-4^					6.5					6.5

**Figure 5:**
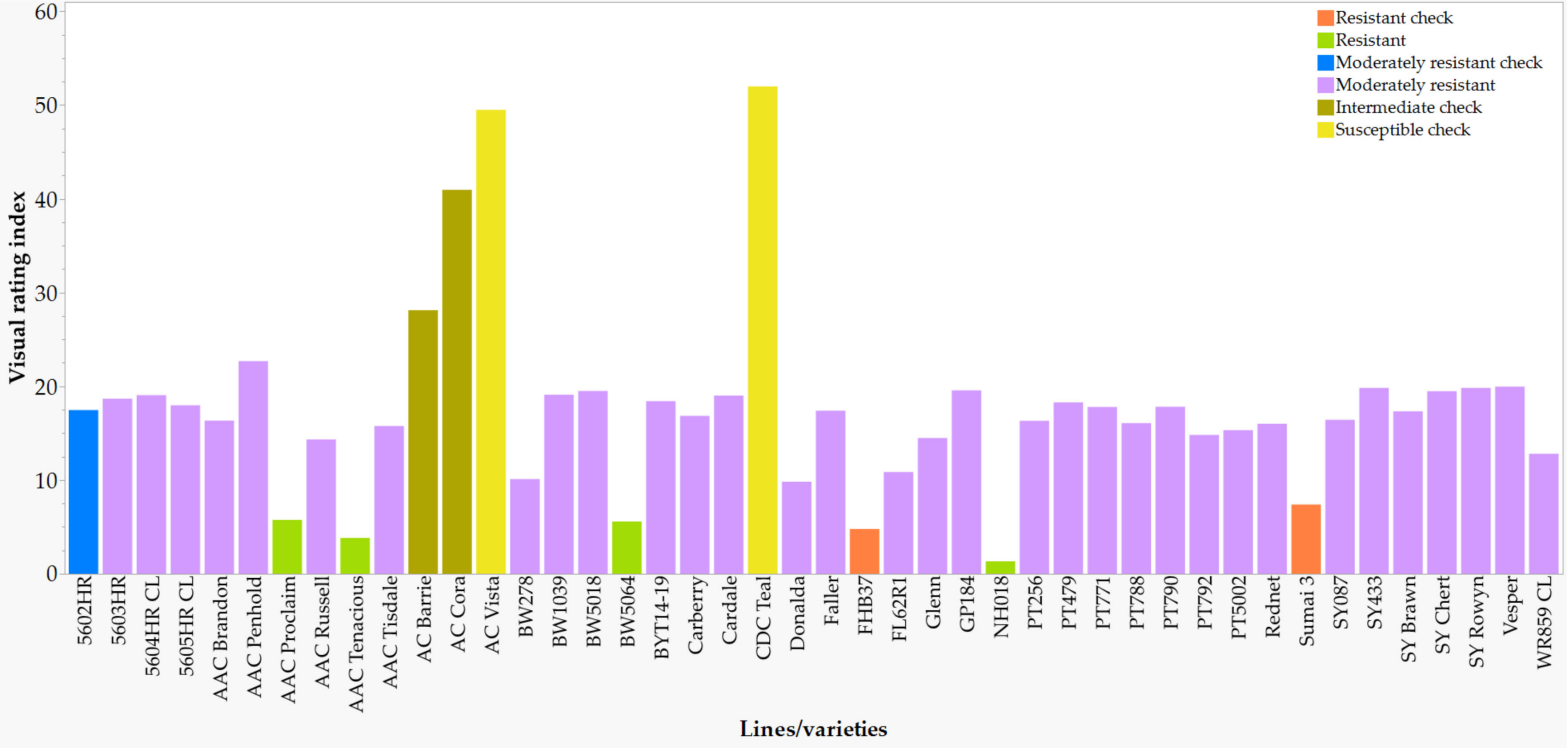
Physical positions of the 28 quantitative trait loci (QTLs) associated with Fusarium head blight (FHB) incidence (black), severity (green), visual rating index (red), flowering time (lavender), and plant height (Skye blue) based on individual environments and all combined environments. The IWGSC RefSeq v2.0 physical map position (Mb) is shown on the left side of the chromosomes, with each horizontal line representing each SNP. QTLs are shown on the right side of each chromosome. See **Supplementary Table S4** for details of SNPs significantly associated with each trait and **Supplementary Figure S4** for details of each QTL. Note the two coincident genomic regions associated with FHB resistance and flowering time on 6A and FHB resistance and flowering time on 7A.

Chromosome and physical positions are based on the International Wheat Genome Sequencing Consortium (IWGSC) RefSeq 2.0. See **Figure 5** and **Supplementary Table S4** for details. PVE refers to the mean phenotypic variance explained by each QTL either in the combined environments or each environment. *The proportion of phenotypic variance explained (PVE) by the QTL based on all combined environments.

Four out of the sixteen FHB resistance QTLs (*QFhb.dms-3B.1, QFhb.dms-5A.5*, *QFhb.dms-5A.7*, and *QFhb.dms-6A.4*) were associated with all three resistance traits (disease incidence, severity, and VRI) in the combined environments and accounted for 5.7–10.2% individually and 27.1-35.6% of the total (cumulative) phenotypic variance (**Table 2**). *QFhb.dms-3B.1* consisted of a cluster of sixteen SNPs, including wMAS000009 (one of the *Fhb1* linked KASP markers widely used for marker-assisted selection), and was mapped at 10.2–23.5 Mb. *QFhb.dms-3B.1* was detected using the overall mean disease incidence, severity, and VRI plus up to three of the seven tested environments (incidence: Carm-2020, Mord-2017, and Mord-2022; severity: Mord-2019; VRI: Carm-2020 and Mord-2019) and accounted for 6.5–10.2% of the phenotypic variances (**Table 2**, **Supplementary Table S4**). Lines and varieties that were homozygous for the resistance alleles at each of the significant SNPs had on average 11.2–30.5% (mean 20.5%), 21.9–33.5% (mean 25.6%), and 33.4–62.9% (mean 41.9%) less disease incidence, severity, and VRI, respectively, than those with the alternative alleles.

*QFhb.dms-5A.*5 was the second QTL associated with disease incidence, severity, and VRI in the combined environments. It consisted of a cluster of 33 SNPs located at 414.2–522.7 Mb and accounted for 7.5–9.2% of the phenotypic variances of disease incidence, severity, and VRI in the individual and combined environments. *QFhb.dms-5A.*5 was a very stable QTL as it was identified not only in the overall means but also up to five out of the seven tested environments for each resistance trait (incidence: Carm-2020, Elora-2017, Mord-2017, Mord-2019, and Mord-2021; severity: Carm-2020, Mord-2017, Mord-2018, and Mord-2022; VRI: Carm-2020, Mord-2017, Mord-2018, Mord-2019, and Mord-2022) (**Supplementary Table S4**). Lines and varieties that were homozygous for the resistance alleles at each of the SNPs for *QFhb.dms-5A.*5 displayed 12.8–18.8% (mean 15.6%), 19.6–32.1% (mean 24.8%), and 27.5–54.5% (mean 38.8%) less disease incidence, severity, and VRI, respectively, than those with the alternative alleles. *QFhb.dms-5A.*7 was the third FHB resistance QTL associated with disease incidence, severity, and VRI in the combined environments plus up to three of the seven tested environments (incidence: Carm-2020 and Mord-2022; severity and VRI: Carm-2020, Elora-2017, and Mord-2022). This QTL was mapped at 683.3–710.1 Mb, consisted of a cluster of 5 SNPs, and accounted for 5.7-9.8% of the phenotypic variances of each trait in the combined and individual environments. Lines and varieties that were homozygous for the resistance alleles at each of the SNPs for *QFhb.dms-5A.*7 showed an average of 7.0%, 10.2%, and 12.6% less disease incidence, severity, and VRI, respectively, than those with the alternative alleles. *QFhb.dms-6A.4* was the fourth resistance QTL associated with disease incidence, severity, and VRI in the combined environments plus up to four of the seven tested environments (incidence: Carm-2020, Mord-2017, Mord-2021, and Mord-2022 plus both severity and VRI recorded in Mord-2022). It consisted of a cluster of 5 SNPs that mapped at 597.6-609.4 Mb and explained 6.7–8.4% of the phenotypic variances of each FHB resistance trait in the individual and combined environments. Lines and varieties that were homozygous for the FHB resistance alleles at each of the significant SNPs for *QFhb.dms-6A.4* showed an average of 25.3%, 35.6%, and 51.6% less disease incidence, severity, and VRI, respectively, than those with the alternative alleles.

*QFhb.dms-2A.2*, *QFhb.dms-2D.*2, and *QFhb.dms-5B.8* were the other FHB resistance QTLs located at 170.8 Mb, 159.1 Mb, and at 640.0 Mb, respectively, which were associated with both disease incidence and VRI recorded in all combined environments. These three QTLs accounted for 6.2-8.0% individually and 20.4–22.2% of the total disease incidence and VRI (**Table 2**). Lines and varieties harboring the FHB resistance alleles showed 7.4–12.2%, 5.1–7.8%, and 1.6–12.9% less disease incidence, severity, and VRI, respectively, than those with the alternative alleles. In the analysis performed on individual environments, however, these three QTLs were less stable because *QFhb.dms-2A.*2 and *QFhb.dms-2D.2* were associated with both disease incidence and VRI recorded in Carm-2020, and incidence in Mord-2017, whereas *QFhb.dms-5B.8* was associated with only disease severity recorded in Mord-2017 (**Supplementary Table S4**).

Seven of the sixteen FHB resistance QTLs identified using the overall means of all combined environments (**Table 2**) were associated with only disease incidence and accounted for 5.9–9.5% individually. These seven QTLs included *QFhb.dms-1A.2* that mapped at 123.9 Mb, *QFhb.dms-2B.*8 (691.1–700.1 Mb), *QFhb.dms-3A.*1 (10.5–26.0 Mb), *QFhb.dms-3D.*1 (3.1–11.7 Mb), *QFhb.dms-5A.*6 (596.2–617.1 Mb), *QFhb.dms-5D.*3 (477.8 Mb), and *QFhb.dms-6D.*1 (3.5–12.0 Mb). Each of these QTLs consisted of a cluster of up to eleven SNPs significantly associated with disease incidence. Lines and varieties that were homozygous for the resistance allele(s) at each QTL showed an average of 23.4–30.5% less disease incidence than those with the alternative alleles. In the GWAS analyses performed on individual environments, *QFhb.dms-2B.8* and *QFhb.dms-5D.3* were associated only with disease incidence recorded at one environment, *QFhb.dms-6D.1* with only disease incidence at three environments, and the remaining four QTLs (*QFhb.dms-1A.2*, *QFhb.dms-3A.1*, *QFhb.dms-3D.1*, and *QFhb.dms-5A.6*) were associated with disease incidence at two environments, disease severity at one environment, and VRI up to three environments (**Supplementary Table S4**).

*QFhb.dms-1A.*1 and *QFhb.dms-7A.*1 were mapped at 12.4–48.4 Mb and 43.0–89.2 Mb, respectively, each consisting of a cluster of 4–5 SNPs, and individually accounted for 6.5– 6.7% of disease severity in the combined environments (**Table 2**). Lines and varieties that harbored *QFhb.dms-1A.1* and *QFhb.dms-7A.1* resistance alleles showed an average of 16.9% and 2.8% less disease severity, respectively, than those with the alternative alleles. In the analyses performed on individual environments, *QFhb.dms-1A.*1 was associated with both disease incidence recorded at Mord-2017 and VRI recorded both at Mord-2018 and Mord-2021, but not disease severity recorded at any of the individual environments. *QFhb.dms-7A.1* was associated with disease incidence recorded at Carm-2020, disease severity recorded at three environments (Elora-2017, Mord-2017, and Mord-2022), and VRI recorded at three environments (Elora-2017, Mord-2017, and Mord-2018) (**Supplementary Table S4**).

*QPht.dms-2A.*3, *QPht.dms-2A.*5, *QPht.dms-6A.2*, and *QPht.dms-7B* were the four QTLs associated with the overall mean plant height recorded in all five combined environments, which were mapped at 679.8–680.0 Mb, 748.8–762.1 Mb, 565.7–615.3 Mb, and 14.9 Mb, respectively. These four QTLs accounted for 6.7–7.5% individually and 28.0% of the total phenotypic variance of plant height recorded in the combined environments (**Table 2**). In contrast to *QPht.dms-7B* which was detected only in the combined environments, the other three plant height QTLs were detected in the over means plus up to three of the five individual environments: *QPht.dms-2A.3* in Mord-2019, *QPht.dms-6A.2* in Mord-2019 and Mord-2022, and *QPht.dms-2A.5* in Mord-2018, Mord-2021, and Mord-2022. The eight flowering time QTLs identified in the combined environments were *QFlt.dms-1A.*4 that was mapped at 579.0 Mb, *QFlt.dms-1B.*2 (675.7 Mb), *QFlt.dms-2A.*1 (511.5 Mb), *QFlt.dms-2A.*2 (760.1–776.7 Mb), *QFlt.dms-2B.*3 (800.7-803.2 Mb), *QFlt.dms-2D* (382.6 Mb), *QFlt.dms-7A.*1 (65.8 Mb), and *QFlt.dms-7B* (578.2 Mb). These QTLs accounted for 6.1–8.3% individually and 57.4% of the total phenotypic variance in the combined environments. In the GWAS analysis performed using BLUEs from individual environments, four out of the eight flowering time QTLs (*QFlt.dms-2A.1*, *QFlt.dms-2D*, *QFlt.dms-7A.1*, and *QFlt.dms-7B*) were not identified in any of the individual environments and the remaining four QTLs (*QFlt.dms-1A.4*, *QFlt.dms-1B.2*, *QFlt.dms-2A.2*, and *QFlt.dms-2B.3*) were detected at one of the three tested environments (**Supplementary Table S4**).

### FHB resistant sources

**Figure 6:**
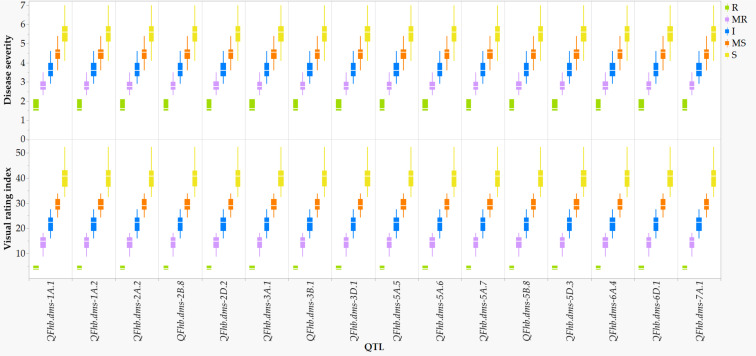
Comparison of the overall Fusarium head blight visual rating index of 198 varieties and lines at each of the 16 disease resistant QTLs: highly resistant (R), moderately resistant (MR), intermediate (I), moderately susceptible (MS), and susceptible (S).

**Figure 7:**
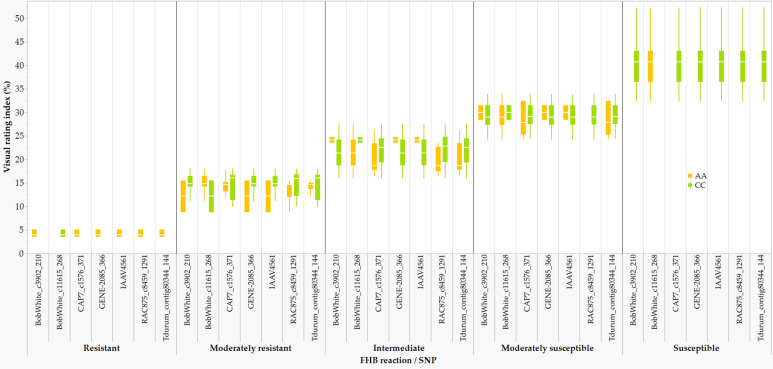
Comparison of the overall Fusarium head blight visual rating index of 198 varieties and lines that were homozygous for AA or CC at the seven SNPs associated with the *QFhb.dms-3B.1*: highly resistant (R), moderately resistant (MR), intermediate (I), moderately susceptible (MS), and susceptible (S).

In comparison with the VRI of the checks computed from all seven combined environments (**Supplementary Table S1**), four of the 249 lines and varieties (AAC Proclaim, AAC Tenacious, NH018, and BW5064) were found to be highly resistant (R), 33 moderately resistant (MR), 60 intermediate (I), 83 moderately susceptible (MS), and 62 susceptible (S). Of the thirty-seven lines and varieties that displayed R and MR (**Figure 5**), twenty-seven belong to the Canada Western Red Spring market class (5603HR, 5604HR CL, 5605HR CL, AAC Brandon, AAC Russell, AAC Tisdale, BW1039, BW278, BW5018, BW5064, BYT14-19, Carberry, Cardale, Donalda, Glenn, PT256, PT479, PT5002, PT771, PT788, PT790, PT792, Rednet, SY433, SY Brawn, SY Chert, and WR859 CL). The remaining ten R and MR lines and varieties were from the Canada Northern Hard Red (Faller, NH018, and Vesper), the Canada Prairie Spring Red (AAC Penhold, AAC Tenacious, and SY Rowyn), and the Canada Western Special Purpose (AAC Proclaim, GP184, and SY087), and an Eastern Canadian spring wheat line (FL62R1). We observed clear differences among the overall visual rating index of the R, MR, I, MS, and S lines and varieties in all sixteen FHB resistant QTLs regardless of the specific linked SNPs (**Figure 6**). Similar trends were observed when the comparisons were made on each SNP linked with the FHB resistant QTLs, which is demonstrated in **Figure 7** using a subset of seven of the sixteen SNPs for *QFhb.dms-3B.*1.

A detailed diversity assessment and relationship of the germplasm used in this study has been published in a previous study (Semagn et al., 2021). To get insight into the genetic relationship of the panel with emphasis on FHB reactions, we computed genetic distance matrices between pairs of varieties/lines from all 26,449 polymorphic SNPs (matrix-1) and 401 SNPs significantly associated with FHB incidence, severity, and VRI in the individual and combined environments (matrix-2). The pairwise distance varied from 0.012 to 0.484 for matrix-1 and 0 to 0.771 for matrix-2 (data not shown), suggesting biased estimates with a decrease in the number of markers. The phylogenetic trees constructed from the distance matrix computed from the 401 SNPs significantly associated with FHB resistance tend to cluster the R and MR lines/varieties better than all SNPs in matrix-1 (**Supplementary Figure S5**). One of the clusters constructed from the 401 SNPs consisted of eleven R and MR cultivars: AAC Penhold, WR859 CL, BW278, Glenn, SY087, Faller, AAC Brandon, AAC Tenacious, AAC Proclaim, FL62R1, and Sumai 3.

## Discussion

### FHB resistance QTLs

The historical and modern Canadian spring wheat varieties and lines used in the present study have been previously used for GWAS to map QTLs associated with agronomic traits and grain characteristics (Semagn et al., 2022b) and resistance to stripe rust, leaf rust, leaf spot, and common bunt (Iqbal et al., 2022). Using the IWGSC RefSeq v2.0 physical information and the overall mean phenotype data of four conventionally and three organically managed environments, we uncovered a total of 108 QTLs associated with days to heading (9), days to maturity (12), plant height (15), lodging tolerance (12), thousand kernel weight (13), test weight (13), grain yield (16), and grain protein content (18). These QTLs accounted for 4.4–11.5% individually and 13.8–73.4% of the total phenotypic variance of each agronomic trait and grain characteristic (Semagn et al., 2022b). For resistance to diseases, we uncovered a total of 37 QTLs associated with the overall means of common bunt (12), leaf rust (13), stripe rust (5), and leaf spot (7), which accounted for 6.6–16.9% individually and 39.4–97.9% of the total phenotypic variance of each disease combined across all environments (Iqbal et al., 2022). The physical positions of *QFhb.dms-1A.1* associated with the combined and individual environments in the present study was mapped 1 Mb away from a QTL previously reported for common bunt resistance (*QCbt.dms-1A.2*) in the same germplasm. *QFhb.dms-4B.3* was about 5.1 Mb away from the stripe rust resistance QTL (*QYr.dms-4B*). The number of lines and varieties used in two previous studies and the current study varied from 192–198, which agrees with the 150–200 population size widely used in QTL discovery studies (Wang et al., 2022). A few examples include 186 durum wheat association mapping panel (Ruan et al., 2020), an association panel of 171 common wheat cultivars (Hu et al., 2020), 187 spring wheat recombinant inbred lines (Poudel et al., 2022), and 171 bread wheat doubled haploid lines (Zhu et al., 2021b).

Flowering time in wheat is controlled primarily by the photoperiod response genes (*Ppd*), vernalization genes (*Vrn*), and “earliness per se”. *Ppd* genes are located on chromosomes 2A (*Ppd-A1*), 2B (*Ppd-B1*), and 2D (*PPd-D1*), while *Vrn* genes are located on 5A (*Vrn-A1*), 5B (*Vrn-B1*), and 5D (*Vrn-D1*) (Laurie, 1997; Worland et al., 1998; Wilhelm et al., 2009; Chen et al., 2013). The physical position of *QFlt.dms-5A.3* (576.3 Mb) associated with flowering time recorded in Mord-2017 was ~11 Mb away from the *Vrn-A1* (*TraesCS5A02G391700*) gene located at 587.4–590.4 Mb depending on the version of the IWGSC RefSeq. Of the five SNPs associated with *QFlt.dms-5B* (553.0–574.4 Mb) in the Mord-2017, the physical positions of both Tdurum_contig32812_325 and BS00022000_51 were 0.6 Mb away from *Vrn-B1* (*TraesCS5B02G396600*) that is located at 573.8–577.1 Mb. Similarly, the position of *QFlt.dms-5D.3* (467.0 Mb) associated with Mord-2017 was 0.1 Mb away from the *Vrn-D1* (*TraesCS5D02G401500*) that is located at 467.1–470.0 Mb. However, the positions of none of the flowering time QTLs discovered in the present study were near the *Ppd-A1*, *Ppd-B1*, and *Ppd-D1* genes.

Flowering time, plant height, and/or anther extrusion/retention have often shown a significant negative correlation with FHB resistance (Miedaner and Voss, 2008; Skinnes et al., 2010; Lu et al., 2011; Lu et al., 2013; Buerstmayr and Buerstmayr, 2016; He et al., 2016; Ruan et al., 2020). For those reasons, the positions of several QTLs associated with FHB resistance overlapped with plant height and/or flowering time QTLs in multiple wheat populations (McCartney et al., 2016; Prat et al., 2017; Ruan et al., 2020; Zhang et al., 2020b). In the combined analysis of all environments, the physical position of FHB resistance QTL on chromosome 7A (*QFhb.dms-7A.1*) overlapped with the flowering time QTL (*QFlt.dms-7A.1*) and another FHB resistance QTL on 6A (*QFhb.dms-6A.4*) overlapped with a plant height QTL (*QPht.dms-6A.2*) (**Table 2**; **Figure 4**; **Supplementary Figure S3**). Liu and colleagues identified eight QTLs associated with five different FHB traits in a biparental population derived from the cross VA00W-38 × Pioneer brand 26R46 of which four coincided with flowering time QTLs (Liu et al., 2012). Colocalization of FHB resistance and plant height QTLs have also been reported on 14 wheat chromosomes (Buerstmayr et al., 2020), which could be due to the pleiotropic effect or tight linkage (Tuberosa et al., 2002; Martinez et al., 2021). The physical positions of the remaining fourteen FHB resistance QTLs identified in the combined environments were different from those QTLs associated with plant height and flowering time.

*QFhb.dms-3B.1* was one of the QTLs that accounted for 6.5–10.2% of disease incidence, severity, and VRI (**Table 2**). *QFhb.dms-3B.*1 is likely the same as the *Fhb1* (syn. *Qfhs.ndsu-3BS*) gene that originated from Sumai 3 (Anderson et al., 2001; Liu and Anderson, 2003) for two reasons. First, the physical positions of *Fhb1* reported in the literature varied from 8 Mb to 21 Mb (Brar et al., 2019a), which overlapped with the 10.2–23.5 Mb interval for *QFhb.dms-3B.*1 (**Table 2**). Second, one of a cluster of SNPs significantly associated with *QFhb.dms-3B.1* in the present study was wMAS000009, which is a KASP marker developed from UMN10_SNP to introgress the *Fhb1* resistance allele through marker-assisted selection (Liu et al., 2008). Lines and varieties that were homozygous for GG at wMAS000009 had on average 18.4%, 34.8%, and 14.3% less disease incidence, severity, and VRI, respectively, than those that were homozygous for AA. Further study in biparental populations is needed to narrow the physical position of *QFhb.dms-3B.1* and determine specific allele(s) present in the Canadian sping wheat panel. Although *Fhb1* is the most consistent and the best source of resistance to a broad spectrum of *Fusarium* species (Anderson et al., 2001; Liu and Anderson, 2003), the phenotypic variance explained by this gene is highly erratic (3–60%) depending on the genetic background (Anderson et al., 2001; Buerstmayr et al., 2002; Bernardo et al., 2012; Wu et al., 2019; Zhang et al., 2020b).

Both *Qfhs.ifa‐5A* (Buerstmayr et al., 2003) and *Qfhi.nau‐5A* (Lin et al., 2006; Xue et al., 2011) have been reported as major FHB resistance QTLs on chromosome 5A and have been given *Fhb5* gene designation. The position of *Fhb5* ranged from 46 Mb to 111 Mb depending on the flanking markers (Brar et al., 2019a). For example, BS00045284_51 located at 110.8 Mb on 5A was one of the SNPs significantly associated with both VRI and disease incidence in two backcross populations derived from CDC Go*4/04GC0139 and CDC Alsask*4/04GC0139. We uncovered two QTLs associated with disease incidence, severity, and VRI (*QFhb.dms-5A.5* and *QFhb.dms-5A.7*) plus a third QTL (*QFhb.dms-5A.6*) located at 414.2–522.7 Mb, 683.3–710.1 Mb, and 596.2–617.2 Mb, respectively (**Table 2**). However, the positions of these three QTLs are far from the *Fhb5* gene. Several other FHB resistance QTLs have also been reported on chromosome 5A, including interspecific populations (Ollier et al., 2020), a durum wheat population derived from Joppa/10Ae564(Zhao et al., 2018a), a RIL population derived from Ningmai 9/Yangmai 158 (Jiang et al., 2020) between IAAV5294 and BS00060445_51 SNP markers. The IWGSC RefSeq v2.0 positions of both IAAV5294 and BS00060445_51 is 503.7 Mb, which overlaps with *QFhb.dms-5A.5* identified in the present study. In a Canadian DH spring wheat population derived from FL62R1/Stettler, a QTL that accounted for 6.3% of FHB resistance has been reported between BS00036839_51 and BobWhite_c2236_111 on 5A (Zhang et al., 2018) located at 397.6–445.4 Mb, which again overlaps with the *QFhb.dms-5A.5*.

Brar et al. (2019a) reported a QTL associated with disease incidence, severity, VRI, and deoxynivalenol (DON) accumulation (*Qfhb.ndwp-6A*) at 602.5–611.8 Mb on chromosome 6A, which accounted for 3.3–9.4% of the phenotypic variance in CDC Alsask/CDC Go near-isogenic lines. The position of *Qfhb.ndwp-6A* overlapped with another QTL reported in a RIL population derived from the cross of ND2603/Grandin population (Zhao et al., 2018b). In the present study, *QFhb.dms-6A.4* was located at 597.6–609.4 Mb (**Table 2**), which overlaps with that of *Qfhb.ndwp-6A* reported in the CDC Alsask/CDC Go and ND2603/Grandin populations. Other studies conducted in wheat have also reported QTLs on chromosome 6A that were associated with disease incidence, severity, and DON accumulation in a winter wheat population derived from NC-Neuse/AGS 2000 (Petersen et al., 2016), disease severity in a winter wheat population derived from Dream/Lynx (Schmolke et al., 2005), and a spring wheat population derived from Surpresa/Wheaton (Poudel et al., 2022).

*QFhb.dms-2A.2* was mapped at 170.8 Mb and was associated with disease incidence and VRI in the combined environments (**Table 2**). Several previous studies reported FHB resistance QTLs on chromosome 2A (Semagn et al., 2007; Zhang et al., 2014; Giancaspro et al., 2016; Sari et al., 2018; Zhang et al., 2018; Gadaleta et al., 2019; Tamburic-Ilincic and Rosa, 2019; Zhang et al., 2020b), including *QFhb.mgb-2A* between IWB43373 (syn. Kukri_c27040_309) and IWB63138 (syn. RAC875_rep_c78744_228) (Giancaspro et al., 2016; Gadaleta et al., 2019). Kukri_c27040_309 and RAC875_rep_c78744_228 are located at 35.0 and 36.5 Mb, respectively (**Supplementary Table S2**), which is more than 134 Mb away from *QFhb.dms-2A.2*. In Canada, FHB resistance QTL on 2A has also been reported at 37–55 Mb in two DH spring wheat populations derived from the cross of FL62R1/Stettler and FL62R1/Muchmore (Zhang et al., 2018; Zhang et al., 2020b), which is also very far from the position of *QFhb.dms-2A.2* (**Table 2**). *QFhb.dms-2D.2* was mapped at 159.1 Mb on chromosome 2D and accounted for 6.9-8.6% of disease incidence and VRI in the combined environments (**Table 2**). FHB resistance QTLs have been reported on chromosome 2D in a winter wheat DH population derived from the cross of Maxine/FTHP Redeemer (Tamburic-Ilincic and Rosa, 2019), in a winter wheat population derived from the cross of Vienna/25R47 (Tamburic-Ilincic and Barcellos Rosa, 2017), and a DH spring wheat population derived from the cross of DH181/AC Foremost (Yang et al., 2005). *QFhb.dms-5B.8* was located at 640.0 Mb and accounted for 6.6–7.2% of the disease incidence and VRI in all combined environments (**Table 2**). FHB resistance QTLs on chromosome 5B have been reported at 448–583 Mb in the FL62R1/Stettler populations (Zhang et al., 2018; Zhang et al., 2020b) and *Qfhs.ndsu-5BL* in durum wheat (Ghavami et al., 2011). *QFhb.dms-5B.8* identified in the present study was, therefore, different from the two FHB resistance QTLs reported on 5B in previous studies.

### FHB resistance sources

Canadian breeders have been able to develop several varieties with moderate-to-intermediate levels of FHB resistance through stepwise accumulations of resistant alleles originated primarily from the Brazilian cv. Frontana and the Chinese cv. Sumai 3 (Gilbert and Tekauz, 2000; Zhu et al., 2019). Frontana has been used as the primary source of resistance in the early stage of Canadian spring wheat breeding, which may have contributed to intermediate level of resistance in Katepwa, AC Barrie, AC Cora, CDC Bounty, Kane, and 5602HR (Gilbert and Tekauz, 2000; McCartney et al., 2016). Sumai 3 has contributed to the release of more than 20 modern varieties, including Cardale, Glenn, Faller, Prosper, AAC Brandon, AAC Elie, Cardale, Carberry, and CDC VR Morris (Zhu et al., 2019). Most varieties with Sumai 3 in their pedigrees displayed moderate-to-intermediate levels of FHB resistance as compared to the intermediate-to-moderate susceptibility of varieties derived from Frontana. Linkage drag is one of the major weakness of heavy dependence on a few exotic FHB resistance sources from Asia and South America, which introduced undesirable genes and QTLs that negatively affect agronomic traits, grain yield, end use quality traits, and susceptibility to other diseases (Brar et al., 2019a).

In the present study, we confirmed and/or identified new FHB resistance sources among the locally adapted spring wheat varieties/lines by evaluating them using the same method and at the same environments. Of the 249 lines/varieties evaluated for FHB reactions, only 2.4% of them displayed a high level of resistance and 13.6% a moderate level of resistance as compared with 60% of them that showed moderate to high levels of FHB susceptibility (**Supplementary Table S1**). We used the VRI of the check varieties/lines with modified threshold values described in previous studies (Yan et al., 2020; Yan et al., 2022) to assign each tested variety/line to the different resistance categories. In contrast to Yan et al. (2020, 2022) who classified entries with a VRI of< 10% into highly resistant and 10-25% as moderately resistant, we considered lines and varieties with a VRI of <7% as highly resistant and those with 10-20% as moderately resistant. The proportion of lines and varieties that were considered highly and moderately resistant agree with a previous study that reported 2.3%, 15.5%, 41.1%, and 41.1% of the wheat samples that expressed high resistance, moderate resistance, moderate susceptibility, and high susceptibility, respectively (Yan et al., 2020). Of a total of 302 Chinese wheat cultivars released from 2005 to 2016, about 96% of them were either moderately susceptible or highly susceptible to FHB and only 4% were moderately resistant to the disease (Ma et al., 2019).

AAC Proclaim (CWSP), AAC Tenacious (CPSR), BW5064 (CWRS), and NH018 (CNHR) were the only varieties that displayed a good level of FHB resistance with an overall VRI of <7%. AAC Proclaim and AAC Tenacious were developed from the cross FHB37/AC Reed (Randhawa et al., 2015) and HY665/BW346 (Brown et al., 2015), respectively, and are characterized by resistance to FHB. Using a DH population derived from the cross AAC Innova/AAC Tenacious, Dhariwal et al. (2020) reported five FHB resistance QTLs that originated from AAC Tenacious at 253.4 Mb on chromosome 2B (*QFhi.lrdc-2B*), at 36.2 Mb (*QFhs.lrdc-2D.1*) and 555.1 Mb (*QFhs.lrdc-2D.2*) on 2D, 279.5 Mb on 5D (*QFhi.lrdc-5D*), and 673.7 Mb on 7A (*QFhi.lrdc-7A*). Comparative histological and transcriptomic analyses performed in AAC Tenacious and a susceptible control (Roblin) following inoculation with *F. graminearum* revealed a restricted infection at the point of inoculation (POI) as compared with a severe infection observed in Robin florets, which was due to a significant cell wall thickening within the rachis node below the POI, and activation of genes putatively involved in cell wall modification and defense response (Nilsen et al., 2021).

Thirty-three varieties and lines expressed a moderate level of VRI based on all seven combined environments (**Supplementary Table S1**) of which twelve (BW278, BW5018, PT256, PT479, PT788, FL62R1, BW1039, BYT14-19, GP184, PT5002, PT790, PT792) were unregistered lines and twenty-one (5603HR, 5604HR CL, 5605HR CL, AAC Brandon, AAC Penhold, AAC Russell, AAC Tisdale, Carberry, Cardale, Donalda, Faller, Glenn, PT771, Rednet, SY433, SY Brawn, SY Chert, SY Rowyn, SY087, Vesper, and WR859 CL) were registered varieties. In previous studies, sixteen of the 25 lines and varieties expressed different levels of resistance to FHB, although they were not simultaneously evaluated for their reaction using the same inoculation method in the same environments. Three of the varieties (5603HR, 5604HR CL, and 5605HR CL) were developed by Syngenta Canada Inc. from a cross of McKenzie//FHB5227/Lars, AC Barrie//Butte86*4/FS4/3/CDC Teal/4/McKenzie/5/(BW288) AC Domain*2/AC Cora, and 99S2232-10 and 99S3228-4, respectively (https://inspection.canada.ca/; accessed 3 May 2023). AAC Brandon was developed from a cross of Superb/CDC Osler//ND744 and expressed moderate resistance to FHB (Cuthbert et al., 2016). AAC Tisdale (PT250) was derived from a cross of Somerset/BW865//Waskada and expressed a moderate level of FHB resistance and low DON accumulation (https://ensqualityseed.com/wp-content/uploads/2021/01/AAC-Tisdale-CWRS.pdf, accessed 23 Feb2023). Carberry was developed from the cross Alsen/Superb and expressed a moderate resistance to FHB (DePauw et al., 2011). Cardale was derived from the cross McKenzie/Alsen and expressed moderate level of resistance to FHB (Fox et al., 2013) originated from Alsen that has Sumai 3 in its pedigree (Zhu et al., 2019). Donalda was developed by the University of Alberta from a cross of Peace/Carberry and displayed an intermediate to moderate levels of FHB resistance (Spaner et al., 2022). Glenn originated from the cross ND 2831/Steele-ND and expressed moderate resistance to FHB (http://ndsuresearchfoundation.org/files/pdf/Ag%20Brochures/Glenn_brochure.pdf; accessed 23 Feb 2023). SY433 (BW433) originated from a cross BW275W/N99-2587 and expressed moderate resistance to FHB (https://inspection.canada.ca/english/plaveg/pbrpov/cropreport/whe/app00008453e.shtml; accessed 23 Feb 2023). WR859 CL was developed from a cross of BW267/3/(97S2199-105-1)AC Barrie//Butte 86*4/FS4 and expressed moderate resistance to FHB (https://inspection.canada.ca/english/plaveg/pbrpov/cropreport/whe/app00007331e.shtml; accessed 23Feb2023). PT771 was developed from the cross of Lovitt//Ning 8331/BiggarBSR/3/BW297 and expressed resistance to FHB originating from Ning 8331 (Spaner et al., 2014). Variety 5604HR CL was developed from the cross AC Barrie//Butte86*4/FS4/3/CDC Teal/4/McKenzie/5/(BW288) AC Domain*2/AC Cora, and has shown moderate susceptibility to FHB (https://inspection.canada.ca/english/plaveg/pbrpov/cropreport/whe/app00007753e.shtml; accessed 23 Feb 2023).

Faller was developed from the cross ND2710/ND688/3/Kitt/Amidon//Grandin/Stoa Sib and expressed intermediate resistance to FHB (https://www.fosterag.ca/seed-products/cereals/31-faller; accessed 23 Feb 2023). Vesper was derived from a cross between female F_1_s (Augusta/Hard White Alpha//3*AC Barrie) and male F_1_s (BW150*2//Tp/Tm/3/2*Superb/4/94B35-R5C/5/Superb), and expressed moderate resistance to FHB (Thomas et al., 2013). AAC Penhold was developed from the cross 5700PR/HY644-BE//HY469 and expressed moderate resistance to FHB (Cuthbert et al., 2017). SY Rowyn was developed from the cross 00S0323-7/N92-0098/99S0051-3-1 and expressed moderately resistant to moderately susceptible to FHB (https://inspection.canada.ca/english/plaveg/pbrpov/cropreport/whe/app00010368e.shtml; accessed 23 Feb 2023). SY087 was developed from the cross between BW361/Alsen and expressed moderate resistance to FHB (https://www.topcropmanager.com/new-cereal-varieties-update-19624/, accessed 23 Feb 2023). FL62R1 is an Eastern Canada hard red spring wheat line derived from a four-way cross involving QG22.24/Alsen//SS Blomidon/Alsen (Comeau et al., 2008; Zhang et al., 2020b). FL62R1 carry both *Fhb1* and *FHb5* genes (Zhang et al., 2018) but expressed different levels of FHB resistance across different studies, including moderate resistance in the present study (**Figure 5**), moderate susceptibility (Zhang et al., 2020b), and high resistance nearly comparable with Sumai 3 (Comeau et al., 2008).

The phylogenetic tree constructed from the 401 SNPs that were associated with FHB resistance in individual and combined environments showed a cluster that consisted of AAC Penhold, WR859 CL, BW278, Glenn, SY087, Faller, AAC Brandon, AAC Tenacious, AAC Proclaim, FL62R1, and Sumai 3 (**Supplementary Figure S5**). AAC Proclaim is related to Sumai 3 in its pedigree because it was derived using FHB37 (HY611/Ning 8331) as one of the parents with Ning 8331 being a Sumai 3 derivative. AAC Tenacious likely carry FHB resistance from one of its moderately resistant progenitor (grandparent) cv. Neepawa (through the male parent BW346) and its FHB-resistant female parent HY665 (Brown et al., 2015). Glenn, Faller, and AAC Brandon inherited FHB resistance from Sumai 3 (Zhu et al., 2019).

All selected varieties and lines are well adapted to western Canada growing conditions and would be valuable resources to further improve the level of FHB resistance through stepwise accumulations of resistant alleles from multiple sources. The University of Alberta breeding program has developed multiple populations using the newly identified resistant sources for selection against *F. graminearum.* Examples include FL62R1/Cardale//5603HR, FL62R1/Cardale//Waskada, PT771/Cardale//Waskada, AAC Tenacious//PT771/Cardale, AAC Tenacious/Cardale, FL62R1/Cardale//Carberry, PT771/Cardale//FL62R1/Cardale, PT771/Cardale//PT588, PT771/Cardale//PT584, PT771/Cardale//Carberry, PT771/Cardale//Cardale, and Cardale//FL62R1/PT584.

## Conclusion

The present study contributed to the identification of new sources of FHB resistance and the associated QTLs in historical and modern Canadian spring wheat varieties and lines. The four varieties and lines that expressed a high level of VRI and the thirty-three varieties and lines that expressed a moderate level of resistance, and the sixteen FHB resistant QTLs provide additional information and data to wheat breeders to develop modern varieties with an enhanced level of FHB resistance in western Canada. Seven of the sixteen FHB resistance QTLs (*QFhb.dms-3B.1*, *QFhb.dms-5A.5*, *QFhb.dms-5A.7*, *QFhb.dms-6A.4*, *QFhb.dms-2A.2*, *QFhb.dms-2D.2*, and *QFhb.dms-5B.8*) are of particular importance as they were associated with disease incidence, visual rating index, and/or disease severity in the overall combined environments and up to five out of the seven tested environments. Two of the sixteen FHB resistance QTLs coincided with flowering time or plant height and the remaining fourteen were physically far from QTLs associated with both agronomic traits.

## Data availability statement

The original contributions presented in the study are included in the article/**Supplementary Material**. Further inquiries can be directed to the corresponding authors.

## Author contributions

KSe, MI, MH, and DS designed the study. KSe analyzed the data and drafted the paper. AB-B and DS acquired funding. MH, KSt, IC, AB-B, and AN evaluated the germplasm. AN’D and CP genotyped the germplasm with the Wheat 90K iSelect array. All authors contributed to the article and approved the submitted version.
