# Supplementary material for: Identification of Fusarium head blight sources of resistance and associated QTLs in historical and modern Canadian spring wheat
Source: Front Plant Sci. 2023 Aug 23;14:1190358. doi: 10.3389/fpls.2023.1190358 (PMC10482112; doi:10.3389/fpls.2023.1190358)
Supplement: Supplementary file 1 [file DataSheet_1.zip › Supplementary Figures 1-5.PDF]

Identification of Fusarium head blight sources of resistance and associated QTLs in historical and modern Canadian spring wheat

Kassa Semagn[1*], Maria Antonia Henriquez[2], Muhammad Iqbal[1], Anita L. Brûlé-Babel[3], Klaus Strenzke[1], Izabela Ciechanowska[1], Alireza Navabi[4,†], Amidou N'Diaye[5], Curtis Pozniak[5], and Dean Spaner[1]*

[1]Department of Agricultural, Food, and Nutritional Science, 4-10 Agriculture-Forestry Centre, University of Alberta, Edmonton, AB T6G 2P5, Canada.

[2]Morden Research and Development Centre, Agriculture and Agri-Food Canada, Morden, Canada.

[3]Department of Plant Science, University of Manitoba, 66 Dafoe Road, MB R3T 2N2, Winnipeg, Canada

[4]Department of Plant Agriculture, Crop Science Building, University of Guelph, Guelph, ON N1G 2W1, Canada

[5]Crop Development Centre and Department of Plant Sciences, University of Saskatchewan, 51 Campus Drive, SK S7N 5A8, Saskatoon, Canada.

*Corresponding authors: fentaye@ualberta.ca; dean.spaner@ualberta.ca

Supplementary Figure S1. Coefficients of determination (R^2) between best linear unbiased estimator (BLUE) and best unbiased predictor (BLUP) values of 249 spring wheat varieties and lines evaluated at seven environments and all combined environments (overall). The environments are the Ian N. Morrison Research Farm in Carman (Carm-2020), the Elora Research Station (Elora-2017), and the Morden Research and Development Center (Mord-2017, Mord-2018, Mord-2019, Mord-2021, and Mord-2022).

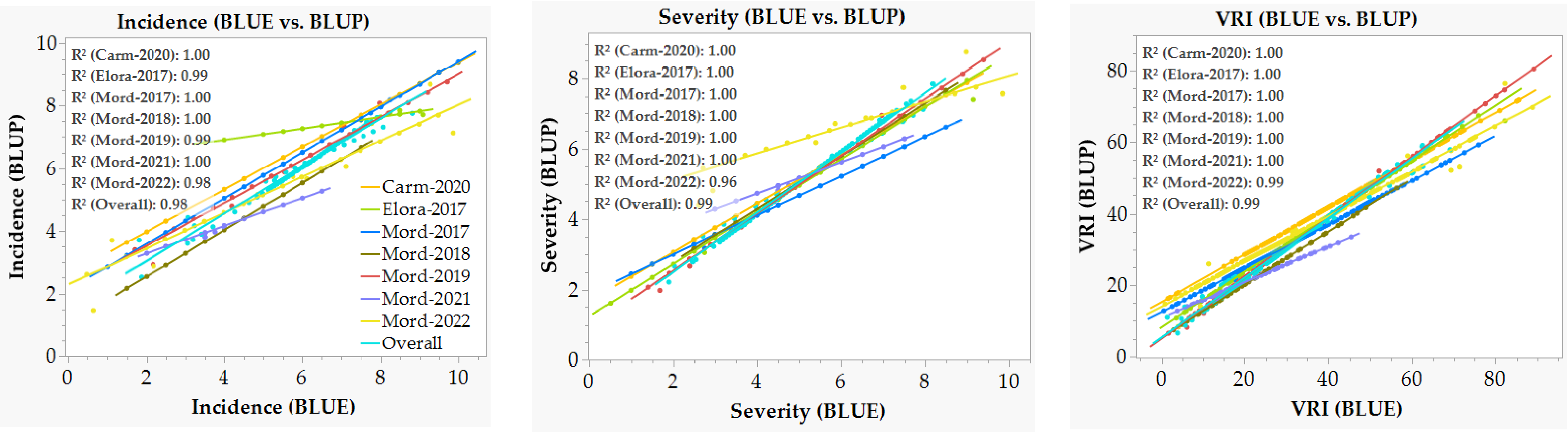

Supplementary Figure S2. Coefficients of determination (R^2) based on the best linear unbiased estimators (BLUEs) of 249 spring wheat varieties and lines computed within each of the seven environment and all environments (overall). The environments are the Ian N. Morrison Research Farm in Carman (Carm-2020), the Elora Research Station (Elora-2017), and the Morden Research and Development Center (Mord-2017, Mord-2018, Mord-2019, Mord-2021, and Mord-2022).

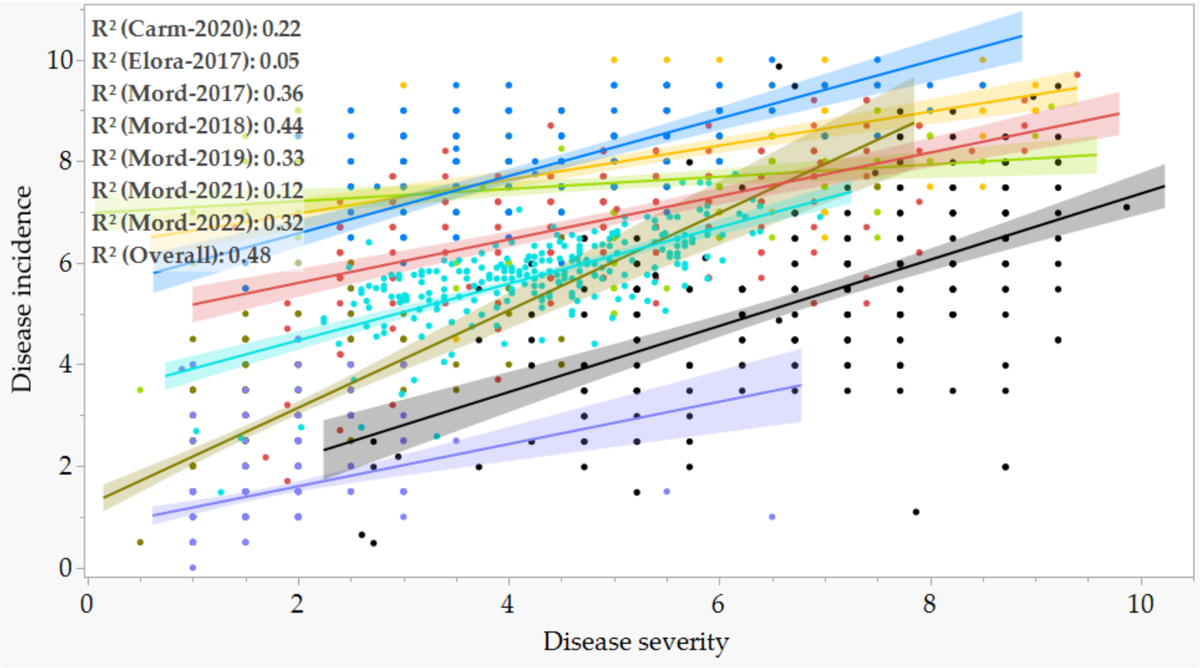

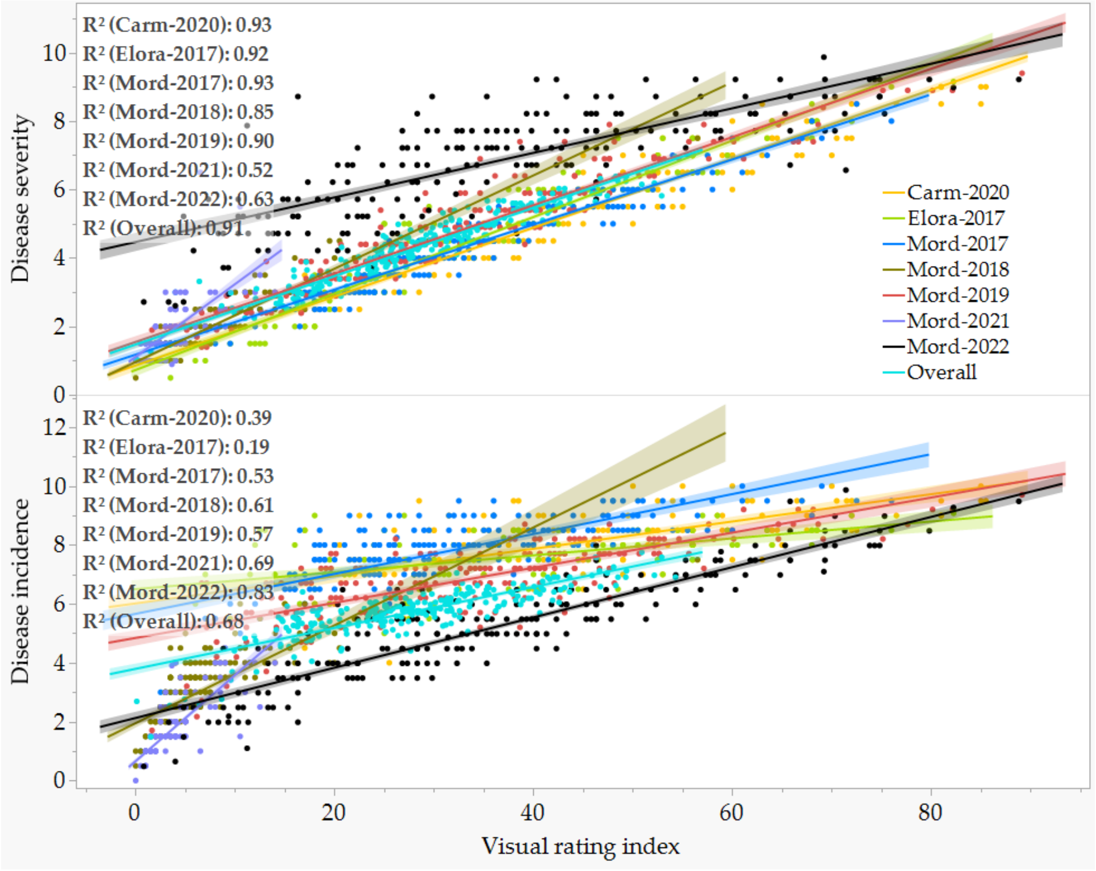

Supplementary Figure S3. A plot of PC_1, PC_2, and PC_3 from principal component analysis of 198 spring wheat varieties and lines by (a) market class and (b) kernel texture and gluten strength. The plots were based on 26,449 polymorphic markers, each with a minor allele frequency of ≥0.05. The market classes are Canada Northern Hard Red (CNHR), Canada Prairie Spring Red (CPSR), Canada Prairie Spring White (CPSW), Canada Western Extra Strong (CWES), Canada Western Hard White Spring (CWHWS), Canada Western Red Spring (CWRS), Canada Western Special Purpose (CWSP), Canada Western Soft White Spring (CWSWS), and known (Ukn). Kernel texture and gluten strength include hard kernel with extra strong gluten (CWES), medium to hard kernel (CNHR), hard kernels (CWRS and CWHWS), medium kernels (CPSR and CPSW), soft kernels (CWSWS), and unknown (Ukn). See Supplementary Table S1 for a list of lines and varieties in each group.

(a) Groups based on market class

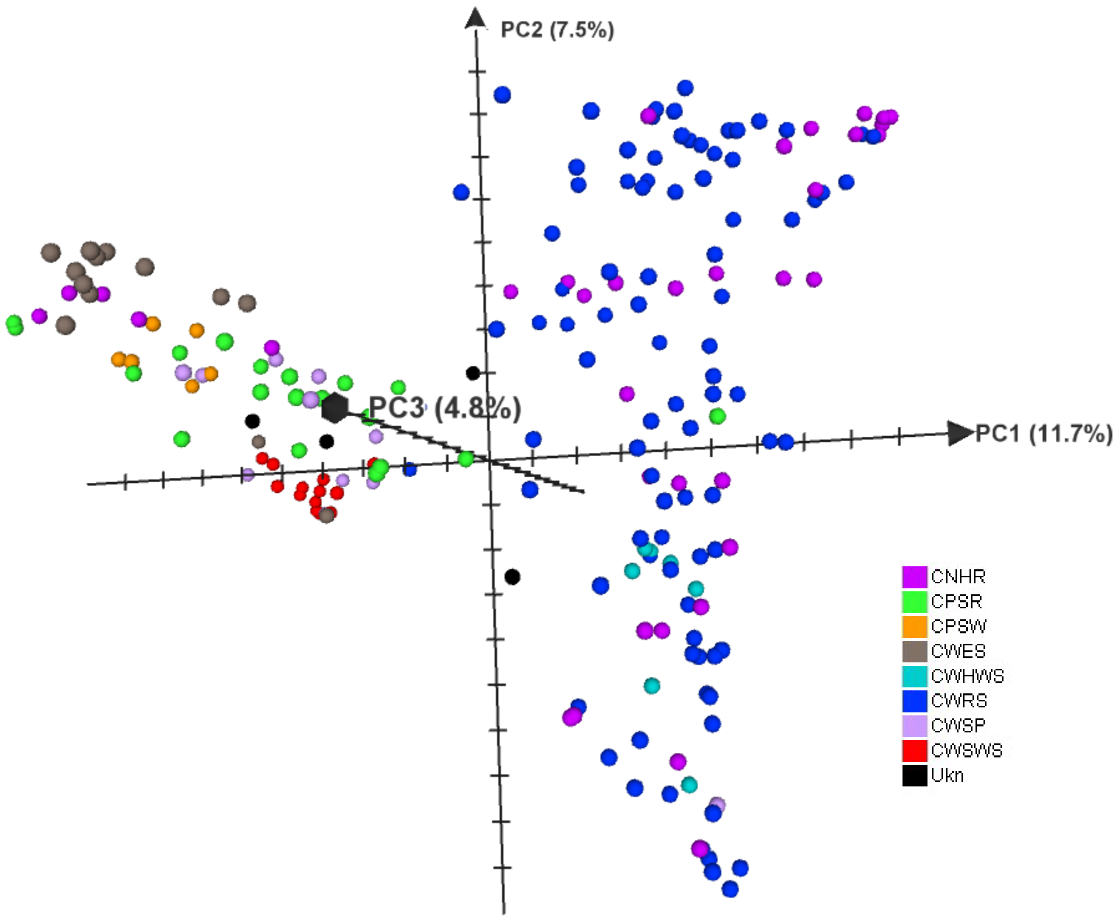

(b) Groups based on kernel hardness/texture and gluten strength

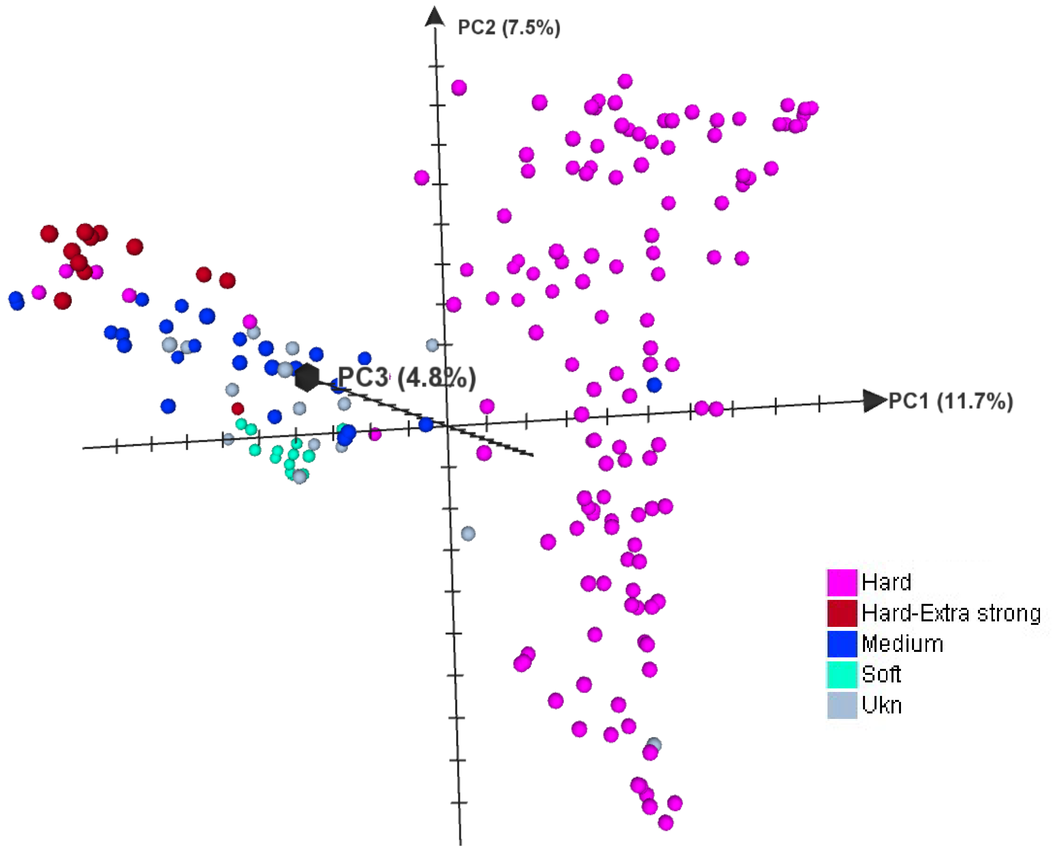

Supplementary Figure S4. Physical positions of the 28 quantitative trait loci (QTLs) associated with Fusarium head blight (FHB) incidence (Inc), severity (Sev), visual rating index (VRI) based on seven individual environments (M: Morden, C: Carmen, E: Elora, followed by trial year, and trait) and overall means (O). QTLs associated with flowering time (FT) and plant height (Pht) were included for comparison purposes. The IWGSC RefSeq v2.0 physical map position (Mb) is shown on the left side of the chromosomes, with each horizontal line representing each SNP. QTLs are shown on the right side of each chromosome. See Supplementary Table S4 for details of SNPs significantly associated with each trait.

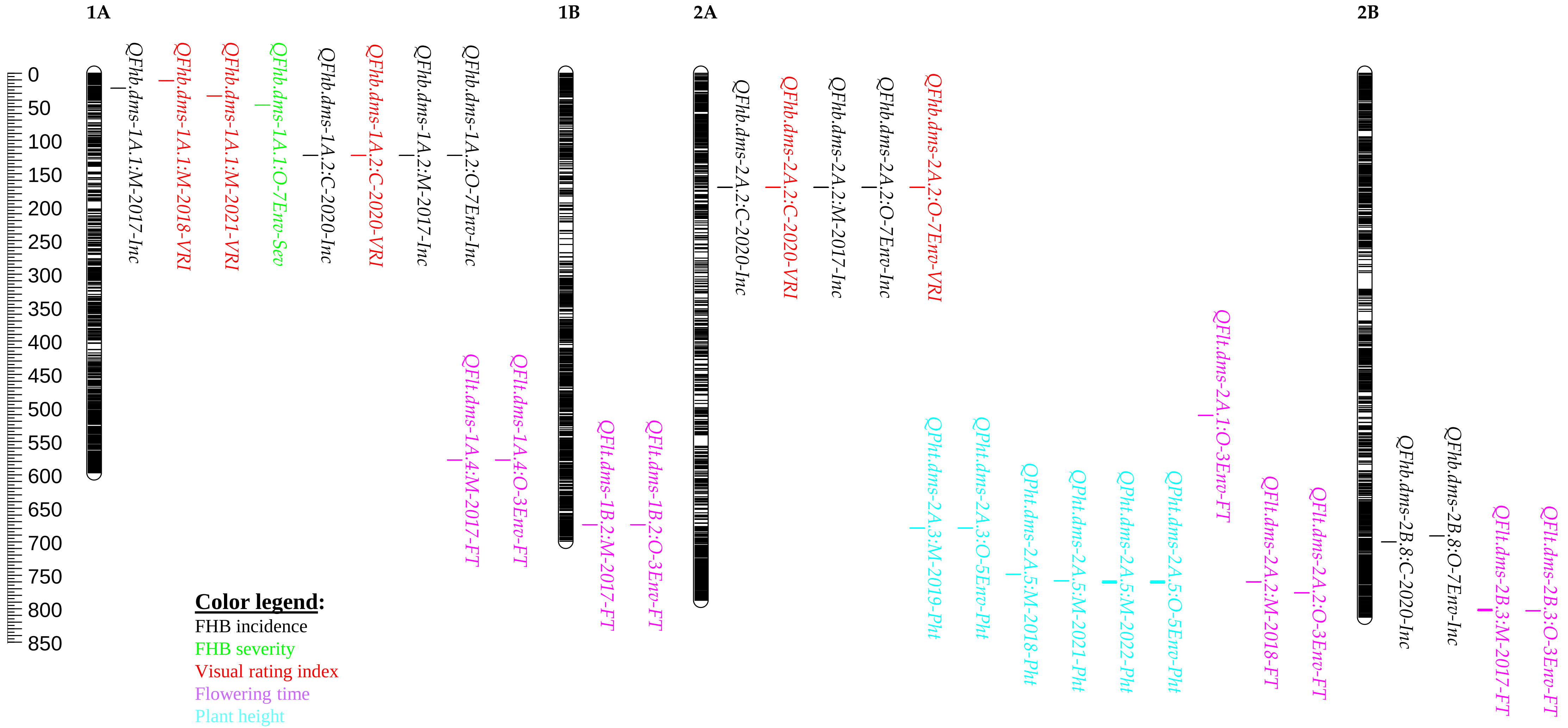

Supplementary Figure S4 (continued)

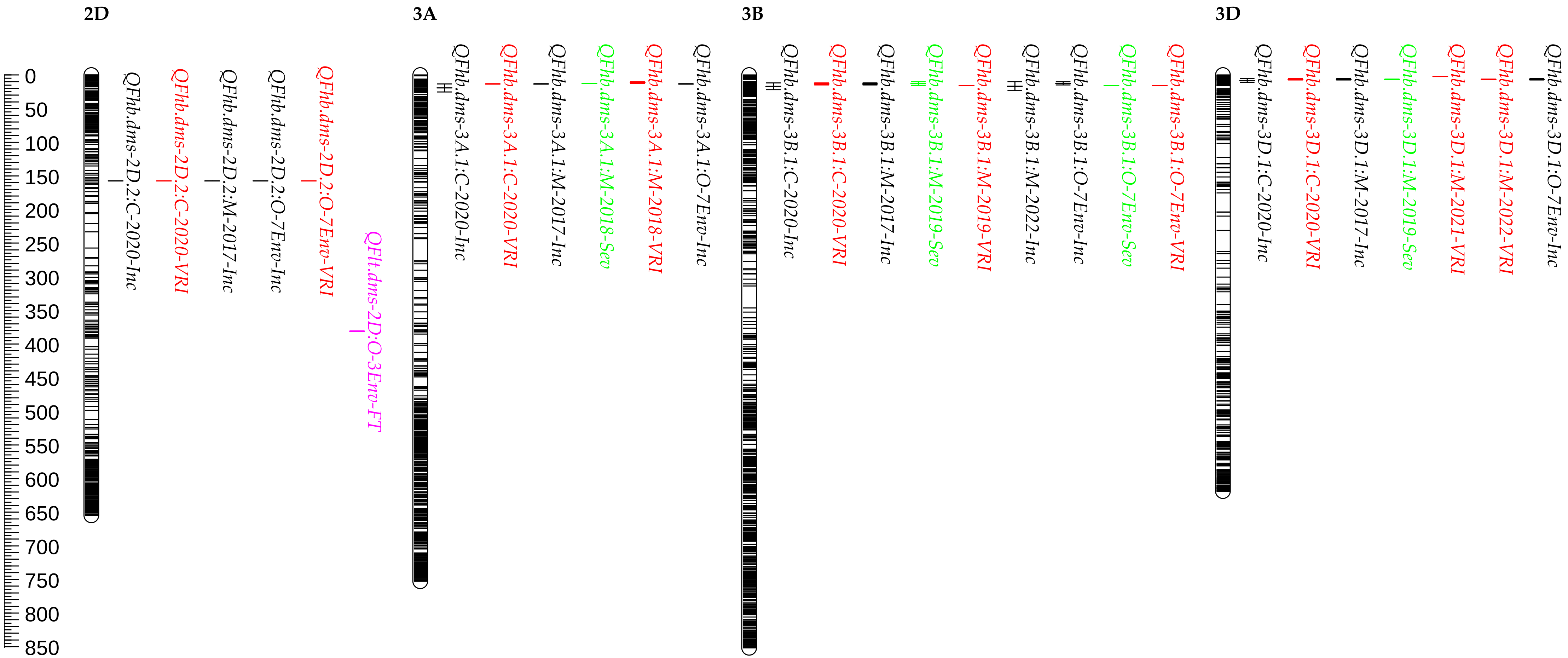

Supplementary Figure S4 (continued)

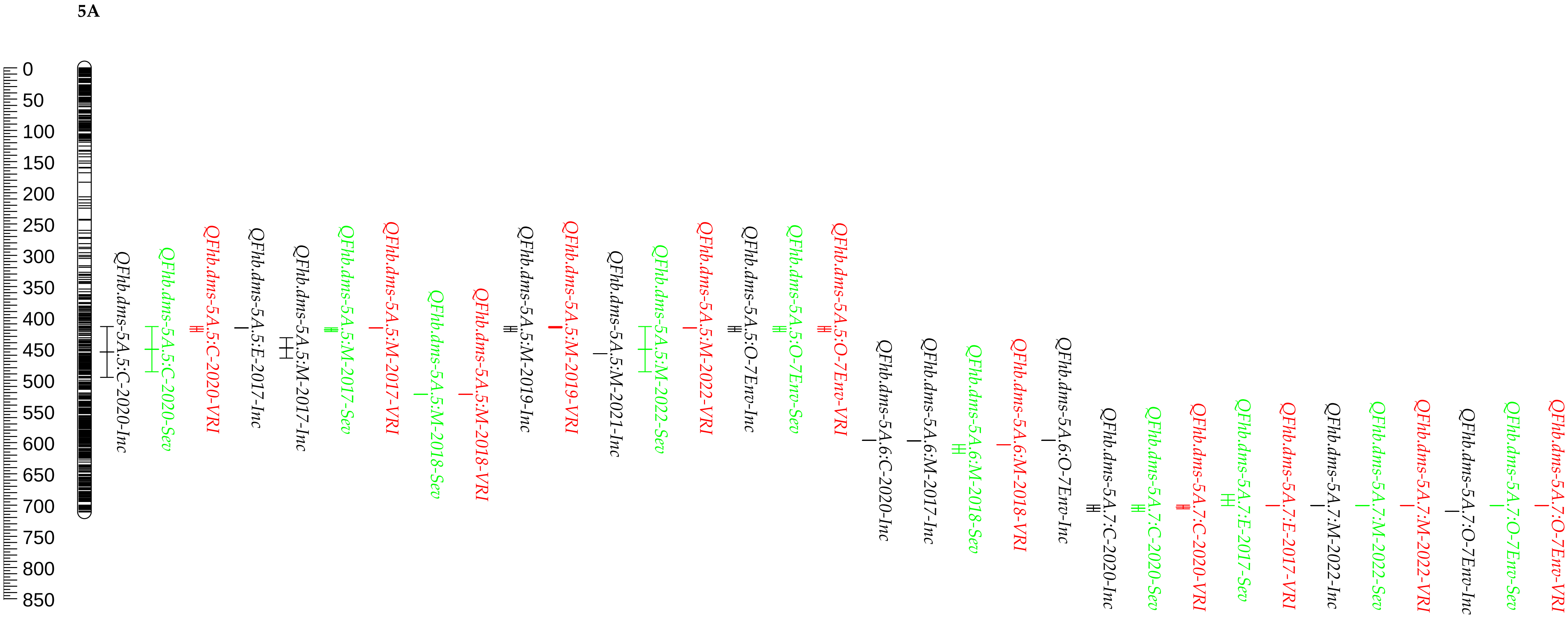

Supplementary Figure S4 (continued)

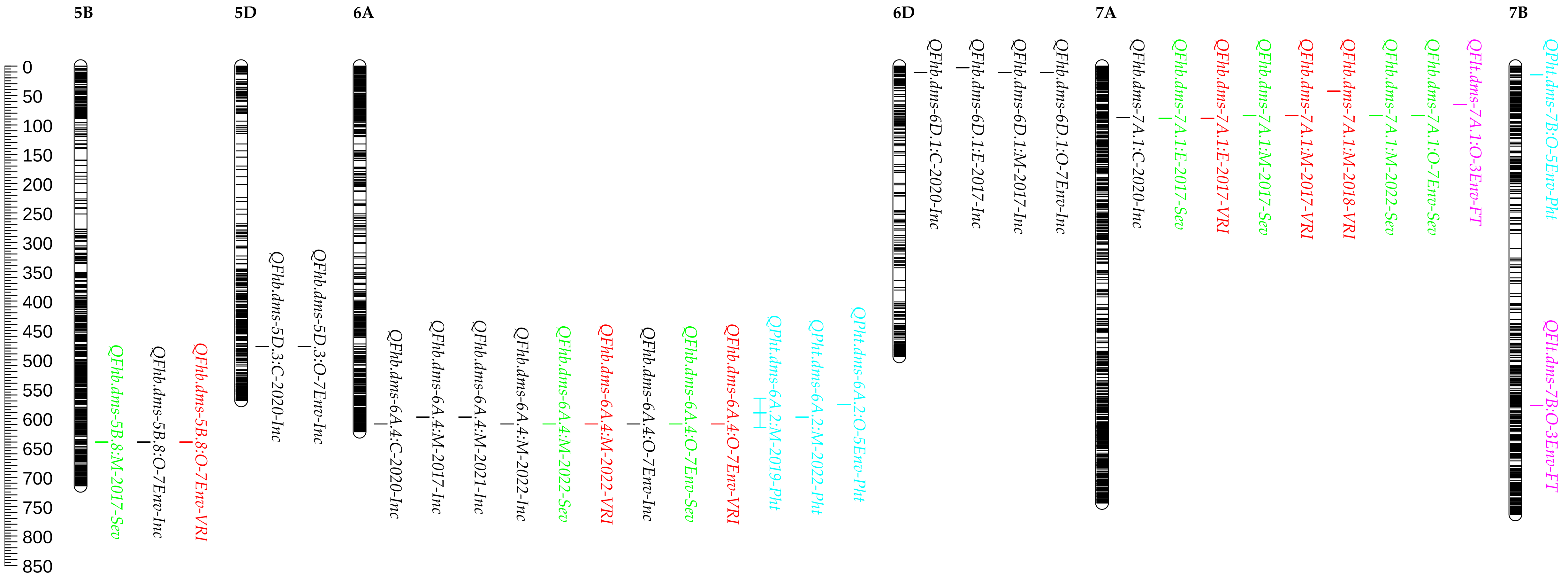

Supplementary Figure S5. Neighbor-joining tree of spring wheat varieties and lines based on identity by state-based genetic distance matrices computed from all 26,449 SNPs and a subset of 401 SNPs significantly associated with Fusarium head blight (FHB) incidence, severity, and visual rating index regardless of the environments. See Supplementary Table S4 for SNPs for details of the SNPs.

(a) Phylogenetic tree of 198 lines and varieties based on all 26,449 SNPs

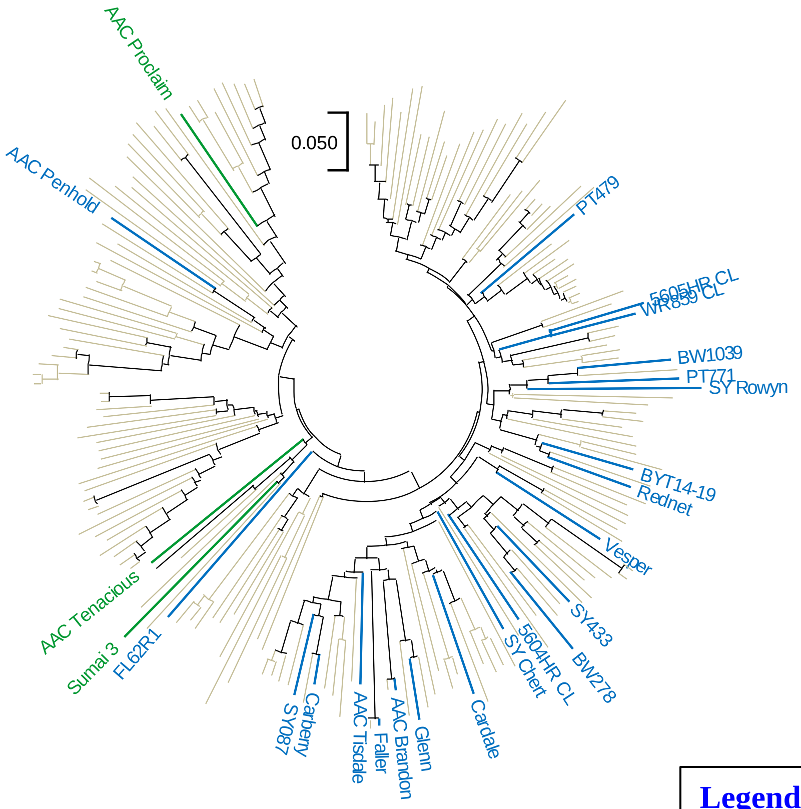

(b) Phylogenetic tree of 198 lines and varieties based on 401 significantly associated with FHB resistance

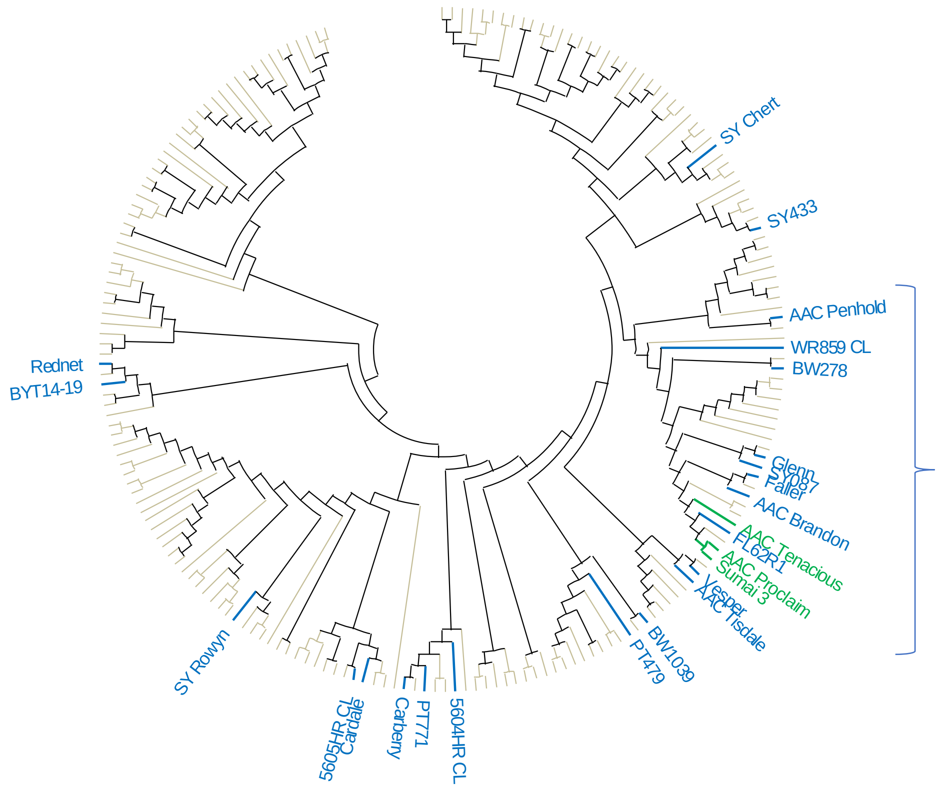

Legend:
Blue: Moderately resistant
Green: Resistant
Intermediate, moderately susceptible, and susceptible
